# Vitamin D and vitamin K1 as novel inhibitors of biofilm in Gram-negative bacteria

**DOI:** 10.1186/s12866-024-03293-6

**Published:** 2024-05-18

**Authors:** Lekaa L. Lutfi, Mona I. Shaaban, Soha Lotfy Elshaer

**Affiliations:** Department of Microbiology and Immunology, Faculty of Pharmacy, Mansoura University, Mansoura, 35516 Egypt

**Keywords:** Gram-negative bacteria, *A. Baumannii*, *K. pneumoniae*, *P. Aeruginosa*, Antimicrobial resistance, Biofilm formation, Vitamin D, Vitamin K1

## Abstract

**Background:**

The persistent surge in antimicrobial resistance represents a global disaster. The initial attachment and maturation of microbial biofilms are intimately related to antimicrobial resistance, which in turn exacerbates the challenge of eradicating bacterial infections. Consequently, there is a pressing need for novel therapies to be employed either independently or as adjuvants to diminish bacterial virulence and pathogenicity. In this context, we propose a novel approach focusing on vitamin D and vitamin K1 as potential antibiofilm agents that target Gram-negative bacteria which are hazardous to human health.

**Results:**

Out of 130 Gram-negative bacterial isolates, 117 were confirmed to be *A. baumannii* (21 isolates, 17.9%), *K. pneumoniae* (40 isolates, 34.2%) and *P. aeruginosa* (56 isolates, 47.9%). The majority of the isolates were obtained from blood and wound specimens (27.4% each). Most of the isolates exhibited high resistance rates to β-lactams (60.7–100%), ciprofloxacin (62.5–100%), amikacin (53.6–76.2%) and gentamicin (65-71.4%). Approximately 93.2% of the isolates were biofilm producers, with 6.8% categorized as weak, 42.7% as moderate, and 50.4% as strong biofilm producers. The minimum inhibitory concentrations (MICs) of vitamin D and vitamin K1 were 625–1250 µg mL-1 and 2500–5000 µg mL-1, respectively, against *A. baumannii* (A5, A20 and A21), *K. pneumoniae* (K25, K27 and K28), and *P. aeruginosa* (P8, P16, P24 and P27) clinical isolates and standard strains *A. baumannii* (ATCC 19606 and ATCC 17978), *K. pneumoniae* (ATCC 51503) and *P. aeruginosa* PAO1 and PAO14. Both vitamins significantly decreased bacterial attachment and significantly eradicated mature biofilms developed by the selected standard and clinical Gram-negative isolates. The anti-biofilm effects of both supplements were confirmed by a notable decrease in the relative expression of the biofilm-encoding genes *cusD*, *bssS* and *pelA* in *A. baumannii* A5, *K. pneumoniae* K28 and *P. aeruginosa* P16, respectively.

**Conclusion:**

This study highlights the anti-biofilm activity of vitamins D and K1 against the tested Gram-negative strains, which emphasizes the potential of these vitamins for use as adjuvant therapies to increase the efficacy of treatment for infections caused by multidrug-resistant (MDR) strains and biofilm-forming phenotypes. However, further validation through in vivo studies is needed to confirm these promising results.

**Supplementary Information:**

The online version contains supplementary material available at 10.1186/s12866-024-03293-6.

## Background

Antimicrobial resistance (AMR) represents a massive threat to public health worldwide. The burden of AMR is tremendous, impacting not only the economic status of patients but also their health and lives [1]. Both counteracting drug availability and patient noncompliance with the use of antimicrobial agents assist in the development of resistance and increasing morbidity and mortality in developing countries [2, 3]. As AMR continues to evolve, last resort antimicrobial agents are now unable to treat many resistant strains reducing the available treatment options [4].

Biofilms consist of communities of microbial sessile (sedentary) cells that attach to abiotic or biotic solid surfaces or air-liquid interfaces. Bacterial biofilms are composed of a matrix of extracellular polymeric substances (EPSs) in which they multiply. This matrix comprises intercellular polysaccharides, proteins and extracellularly released DNA [5, 6]. Mature biofilm-embedded cells are a thousand times more resistant to antibiotics than their planktonic counterparts are. This is due to the limited penetration of antimicrobial agents through the biofilm matrix and the ability of biofilm cells to upregulate efflux pumps and other resistance genes [7]. Hence, within the human body, this complex microbial biofilm provides an environment preventing host immune defences and inactivating a massive number of conventional antimicrobial agents, leading to treatment strategy failure and prolonged hospitalization in clinical settings [8].

*Acinetobacter baumannii* (*A. baumannii*), *Klebsiella pneumoniae* (*K. pneumoniae*) and *Pseudomonas aeruginosa* (*P. aeruginosa*) are the most common Gram-negative bacteria and have high biofilm abilities and multiple resistance to different antibiotic classes according to the World Health Organization (WHO) ranking of multi-drug resistant (MDR) pathogens [9]. Additionally, *A. baumannii, K. pneumoniae* and *P. aeruginosa* are members of a bacterial pathogenic group called ESKAPE (*Enterococcus faecium*, *Staphylococcus aureus*, *K. pneumoniae*, *A. baumannii*, *P. aeruginosa* and *Enterobacter spp*), which have the ability to escape the microbicidal action of antimicrobial agents, express an arsenal of virulence factors, invade the immune system and cause life-threatening infections, particularly in immunocompromised individuals [10, 11]. Severe health complications include bacteraemia, ventilator-associated pneumonia, urinary tract infection, skin and soft tissue infections, neonatal sepsis, and complicated intra-abdominal and burn infections [12].

Approximately 65% of bacterial infections and 80% of chronic nosocomial diseases are attributed to biofilm impenetrability and subsequent drug resistance especially on biological tissue surfaces and medical devices such as respiratory supports and catheters [13]. Therefore, there is an urgent need to discover new approaches to combat *A. baumannii, K. pneumoniae* and *P. aeruginosa* biofilm-related infections since the traditional and even newer antibacterial agents are insufficient for eradicating colonized bacterial infections.

The application of vitamins in treating MDR biofilms is receiving increased amount of attention. For instance, vitamin K3 (menadione)-coated sutures have been shown to be effective as powerful biofilm inhibitors against staphylococcal-associated surgical site infections [14]. In addition, ascorbic acid (vitamin C) has shown promising activity as an antibacterial and antibiofilm agent against carbapenem-resistant hypervirulent *K. pneumoniae, P. aeruginosa, Proteus mirabilis* and uropathogenic *E. coli* [15–18]. A significant reduction in biofilm density caused by a wide panel of human pathogens was also verified through the use of vitamin E [19]. Vitamin D is a fundamental supplement obtained either from the diet or by skin synthesis upon exposure to sunlight. It aids in the absorption of many minerals including magnesium, iron, zinc and phosphate which are responsible for the production of rigid protective tissues such as dental enamel. Vitamin D is linked to the disruption of dental plaque, a naturally occurring biofilm [20]. Furthermore, many reports have shown a significant correlation between lower serum vitamin D levels and chronic dental caries in children [21–23]. Vitamin D has also aided in fighting bacterial infections caused by *S. aureus* [24], *P. aeruginosa* [25] and *Helicobacter pylori* [26] either through triggering macrophage-mediated clearance or by reversing the efflux system, thereby restoring antibiotic activity. However, no studies have been performed to assess the effect of vitamin D (cholecalciferol) or vitamin K1 (phytomenadione/phylloquinone) on biofilm formation. Therefore, the present study aims to evaluate the activity of WHO-approved vitamin D and vitamin K1 as potential novel therapies to combat biofilm formation in Gram-negative organisms. The impact of both vitamins on bacterial attachment and mature biofilms was examined. Additionally, their effects on the expression levels of the biofilm regulatory genes in the tested isolates were assessed.

## Methods

### Bacterial strains

A total of 130 Gram-negative isolates of *Acinetobacter, Klebsiella* and *Pseudomonas* were clinically isolated from different sources such as blood, wounds, urine, bronchoalveolar lavage (BAL), sputum, burns and ear swabs. All specimens were obtained from the Al-Qasr Al-Aini Hospital, Cairo Hospital and Mansoura University Hospital and handled with the approval of the Research Ethics Committee, Faculty of Pharmacy, Mansoura University. The bacterial standard strains *A. baumannii* (ATCC 19606 and ATCC17978), *K. pneumoniae* (ATCC 51503), and *P. aeruginosa* (PAO1 and PAO14) were also included in this study. Based on microbiological laboratory standards, all the isolates were identified colony morphology on MacConkey’s agar media (Oxoid, UK). Reddish-pink colonies of *Acinetobacter* were selectively picked from chromogenic CHROM™ agar (Pioneer, France, Paris). Green *P. areugionsa* colonies were picked from cetrimide agar. The IMVic (indole, methyl red, Voges-Proskauer, and citrate utilization) test was also conducted for further confirmation of *Klebsiella* and *Pseudomonas* isolates [27]. The purified isolates were preserved in a 25% v/v glycerol stock at -80 °C.

**Molecular detection of *A. baumannii *clinical isolates**.

Multiplex polymerase chain reaction (multiplex PCR) was carried out to detect *recA* gene (characteristic of the *Acinetobacter* genus) and *16–23 S rRNA* gene intergenic spacer (*ITS*) region which is specific for *A. baumannii spp* [28]. In this regard, genomic DNA was first extracted by suspending fresh bacterial colonies in sterile nuclease-free water, after which the PCR tubes were boiled for 10 min at 95 ℃ [29, 30]. The PCR included the following cycling procedure: 12.5 µl of 2X Dream Taq™ Green PCR Master Mix (Thermo Scientific, USA), 0.5 µl of each forward and reverse primer (Table 1), and 1 µl of extracted DNA. The volume was adjusted with nuclease-free water to a final volume of 25 µl. The addition of nuclease-free water instead of bacterial lysate to the other PCR mixture served as a negative control. The cycling procedure consisted of initial denaturation at 94 ℃ for 3 min; 35 cycles of denaturation at 94 ℃ for 30 s and annealing for 30 s at 60 ℃ and termination of the reaction by a final extension step at 72 ℃ for 10 min. After staining with ethidium bromide, two bands at the correct predicted size for *recA* (425 bp) and *16–23 S rRNA ITS* (208 bp) were visualized on an agarose gel (1.5% w/v) which indicated positive *A. baumannii* isolates.

**Table 1:** Sequences of primers used in the study

Organism	Primer	Sequence (5′→3′)	AT (°C)	Amplicon size (bp)	Reference
***A. baumannii***	P-rA1 F	CCTGAATCTTCTGGTAAAAC	50	425	[28]
	P-rA2 R	GTTTCTGGGCTGCCAAACATTAC			
	P-Ab-ITS F	CATTATCACGGTAATTAGTG		208	[28]
	P-Ab-ITS R	AGAGCACTGTGCACTTAAG			
	CusD F	AGTCACAACATCGGTCCCAT	56	193	[110]
	CusD R	AAGTTCGGTGCGTCCTTCTA			
	RpoB Ac F	ACAAAGTAATGCGTCCAGGC	55	121	[111]
	RpoB Ac R	CGGTTGAACTTCATACGACCT			
***K. pneumoniae***	BssS F	GATTCAATTTTGGCGATTCCTGC	60	225	[112]
	BssS R	TAATGAAGTCATTCAGACTCATCC			
	RopD F	AAGACGAAGATGAAGACGCC	57	129	This study
	RopD R	CTTTGGCTTTGATGGTGTCG			
***P. aeruginosa***	PelA F	AAGAACGGATGGCTGAAGG	58	148	[113]
	PelA R	TTCCTCACCTCGGTCTCG			
	RopD F	CGAACTGCTTGCCGACTT	56	131	[114]
	RopD R	GCGAGAGCCTCAAGGATAC			

### Antimicrobial susceptibility testing

The antimicrobial susceptibility of all the purified *A. baumannii, K. pneumoniae* and *P. aeruginosa* isolates was assessed using the Kirby-Bauer disc diffusion method [31] according to the Clinical Laboratory Standard Institute (CLSI) [32]. Pure bacterial cultures were adjusted to match the 0.5 McFarland standard and streaked on sterile Mueller-Hinton agar plates where eight antimicrobial agents (Oxoid, UK) from different classes were applied as follows: amoxicillin/clavulanic acid (AMC, 20/10 µg), cefotaxime (CTX, 30 µg), ceftazidime (CAZ, 30 µg), cefepime (FEP, 30 µg), imipenem (IPM, 10 µg), amikacin (AK, 30 µg), gentamicin (CN, 30 µg) and ciprofloxacin (CIP, 10 µg). The resultant inhibition zones around each disc were measured and the results were determined to be sensitive, intermediate and resistant in accordance with CLSI charts (32).

### Detection of biofilm formation

Biofilm formation of all clinical isolates was assessed using a flat-bottom 96-well polystyrene microtiter plate assay [33, 34]. *A. baumannii, K. pneumoniae* and *P. aeruginosa* isolates were propagated overnight in tryptic soy broth (TSB) at 37 °C with shaking at 200 rpm. The wells were filled in sextuplicate with 200 µl of each bacterial culture diluted to the 0.5 McFarland standard. Negative control wells seeded with 200 µl of TSB only were also included. The plates were incubated overnight at 37 °C, after which the bacterial cultures were gently aspirated to remove planktonic cells, followed by washing well with 250 µl of sterile physiological saline. The formed biofilms were fixed with 250 µl of methanol for 15 min and stained with 250 µl of 1% w/v crystal violet (CV) for 20 min. The excess crystal violet was removed, and 250 µl of 33% w/v glacial acetic acid was added to dissolve the stained biofilms. The optical density of each well was measured by microplate ELISA reader at 570 nm. The isolates were classified into four categories as indicated: OD ≤ ODc, nonadherent (nonbiofilm producer); ODc < OD ≤ 2 ODc, weakly adherent (weak biofilm producer); 2 ODc < OD ≤ 4 ODc, moderately adherent (moderate biofilm producer); and 4 ODc < OD, strongly adherent (strong biofilm producer), where ODc (cut-off OD) was defined as 3 standard deviations above the mean OD of the negative control [34, 35].

### Evaluation of the minimum inhibitory concentrations (MICs) of vitamin D and Vitamin K1

The minimum inhibitory concentrations (MICs) of vitamin D and vitamin K1 were determined using 96-well flat bottom microtiter plates according to the CLSI broth microdilution protocol [32]. The MICs of both vitamins were evaluated against selected Gram-negative isolates known for strong biofilm production, including *A. baumannii* standard strains (ATCC 19606 and ATCC 17978) and clinical isolates (A5, A20 and A21), *K. pneumoniae* standard strains (ATCC 51503) and clinical isolates (K25, K27 and K28), in addition to *P. aeruginosa* PAO1 and PAO14 standard strains and P8, P16, P24 and P27 clinical isolates. The Muller-Hinton broth (MHB) was used to propagate the tested isolates overnight at 37 °C, after which the cultures were diluted to a final concentration of 1 × 10^6^ CFU/ml. One hundred microliters of injectable vitamin D (Memphis Company) (1.25 mg/ml) and vitamin K1 (Amoun Pharmaceutical Co. S.A.E.) (10 mg/ml) were prepared as twofold serial dilutions with MHB in 96-well microtiter plates. Then, 10 µl of the diluted bacterial suspension was added to each well to reach a final concentration of 10^5^ CFU ml^− 1^ and the plates were incubated at 37 °C under aerobic conditions for 24 h. Nontreated bacteria and MHB broth medium were used as positive and negative controls, respectively. The MICs were calculated as the lowest concentration of vitamin D or K1 that inhibited bacterial growth and sub-MICs (1/2 MIC and 1/4 MIC) were calculated for further experiments.

### Effect on cell growth kinetics

The effect of vitamin D and vitamin K1 on the growth of three bacterial isolates, *A. baumannii* A5, *K. pneumoniae* K28 and *P. aeruginosa* P16, was estimated by direct optical density measurements. A single pure colony of each isolate was inoculated in Luria-Bertani (LB) broth and shaken (150 rpm) at 37 °C. The overnight culture was inoculated (1 × 10^6^ CFU/ml) into two preparations: untreated and treated with a 1/2 MIC of each tested vitamin. All the preparations were incubated at 37 °C with agitation at 120–150 rpm and the sample (200 µl) was aspirated at 2 h intervals for 24 h, after the bacterial growth was at OD600 nm via ELISA spectrophotometer (BioTek, USA).

### Effect of vitamins on cell viability

The viable bacterial cell number was evaluated by counting the colony forming units (CFUs) before and after treatment with 1/2 MICs of vitamins D and K1. At each time interval from the previous procedure, tenfold serial dilution in LB broth was performed for each suspension. Agar plates were prepared and divided into four quadrants. Five microliter from each dilution in the series was applied in each corner per quadrant. The plates were kept at room temperature until complete inoculum drying and incubated in an inverted position at 37 °C for 24 h. Viable cells were calculated in terms of CFUs/ml according to the following formula: CFU/ml= (average number of colonies x dilution factor)/ the inoculum volume on agar plate (ml). The data are expressed as log CFU/ml.

### Antibiofilm activities of vitamin D and vitamin K1

#### Inhibition of initial bacterial attachment

The effect of vitamin D and vitamin K1 on bacterial adherence and initial biofilm configuration was evaluated using the crystal violet (CV) technique [36, 37]. Both standard and strong biofilm-producing clinical isolates, as previously selected, were propagated in TSB at 37 °C. The next day, each bacterial culture was diluted to 0.5 McFarland turbidity and treated with each vitamin at sub-MICs (1/2 MIC and 1/4 MIC). The wells of the microtiter plate were seeded with untreated and treated cultures (200 µl each), including negative control wells containing TSB only. The plates were incubated overnight at 37 °C. The biofilm-forming ability of the tested isolates was determined with a CV assay, as described subsequently.

### Mature biofilm inhibition assay

The effects of vitamins D and K1 were estimated against preexisting biofilms of standard strains and previously identified clinical isolates, which were powerful biofilm producers. On flatted-bottom 96-well polystyrene plates, 200 µl of each tested isolate (final cell concentration = 1.5 × 10^8^ CFU mL^− 1^) was added to each well. Negative control wells containing only TSB were also included. After overnight incubation at 37 °C to allow biofilm maturation, unattached planktonic cells were aspirated, and the wells were rinsed twice with saline solution. Two hundred microliters of sterile TSB was added to the negative control wells, and another TSB mixture supplemented with 0.5×, 1×, and 2× the MIC of both vitamin D and K1 was added to the conformable wells. The plates were then reincubated at 37 °C for 24 h, and the effect of variable concentrations of both vitamins on the mature biofilm was detected using CV assays, as described below [36].

### Quantification of biofilms by crystal violet (CV) assay

Following static overnight incubation of the plates with and without varying concentrations of vitamins, the planktonic cells were aspirated, leaving the adhered biofilm, which was washed twice and fixed with methanol (250 µl) for 15 min. After removal of excess methanol, the plates were allowed to dry before biofilm staining with CV (1% w/v). Excess dye was gently rinsed under tap water, and the adhered biofilms were quantified through solubilization in 250 µl of glacial acetic acid (33% v/v). The absorbance was further measured at 570 nm using a microtiter plate reader, and the percentage of biofilm formation in vitamin D- and K1-treated cultures was calculated compared to that in unchallenged cultures [38, 39].

### Genotypic analysis

Three housekeeping genes, *rpoB*, * ropD* and * ropD*, in addition to three other biofilm-related genes, *cusD*, *bssS* and *pelA*, specific for *A. baumannii* (A5), *K. pneumoniae* (K28) and *P. aeruginosa* (P16), respectively were investigated by PCR. As previously mentioned, genomic DNA was extracted, and simplex PCR was performed in a 25 µl reaction mixture using the primers and annealing temperatures detailed in Table 1. The PCR program included an initial denaturing cycle at 95 °C for 5 min, followed by (denaturation at 95 °C for 30 s, annealing for 30 s and extension at 72 °C for 1 min) for 35 cycles and a final extension cycle at 72 °C for 5 min. Agarose gel electrophoresis (1.5% w/v) was used to visualize the successful amplification of the PCR product according to the size of the amplicon, as demonstrated in Table 1, and the gel images were captured using a gel documentation system (Model Gel Documentation 1.4, 1189, AccuLab, New York, USA).

### Real-time PCR assay

The effect of vitamin D and vitamin K1 on the expression of the biofilm genes *cusD*, *bssS* and *pelA* was assessed through real-time polymerase chain reaction (RT-PCR). The clinical isolates A5, K28 and P16 were cultivated under the same conditions in the presence and absence of (1/2 MIC) both vitamins. Cultures were subjected to shaking at 150 rpm for 5–6 h until the logarithmic phase of growth was reached. Next, the cultures were centrifuged at 4 ℃ for 20 min at 8000 rpm to collect the bacterial cells, and total RNA was extracted using TRIzol reagent (Oxoid, Basingstoke, Hants, UK) according to the manufacturer’s instructions. cDNA was synthesized using a Thermo Scientific RevertAid First Strand cDNA Synthesis Kit. In addition, the concentration and purity of the extract were estimated using a Nanodrop (BioDrop, UK). Any contaminating DNA was removed using DNase enzyme (Enzymonics, Korea) according to the manufacturer’s instructions, followed by precipitation of pure mRNA with an equal volume of isopropanol and double washing with ethanol (75% v/v). After complete removal of the ethanol, the mRNA pellets were resuspended in 20 µl of RNase/DNase-free water (Sigma Aldrich, UK).

RT-PCR analysis was performed using HERA Plus SYBR Green on a Rotor-Gene Q thermocycler (Qiagen, Valencia, CA, USA) utilizing the biofilm-specific primers listed in Table 1. The relative expression levels of *cusD*, *bssS* and *pelA* genes were normalized to the expression levels of *rpoB*, * ropD*, and * ropD* housekeeping genes in *A. baumannii K. pneumoniae* and *P. aeruginosa*, respectively, in accordance with the 2^−∆∆CT^ analysis method [40]. Comparison of gene expression, in terms of fold change, between untreated and vitamin-treated cultures was also performed.

### Statistical analysis

An Excel sheet was used to calculate the average and standard deviation of each experiment, which was conducted in triplicate. GraphPad Prism software (version 5.00) was used for all the statistical analyses. The chi-square test was utilized for intergroup comparisons in addition to comparisons between treated and untreated control isolates cultivated under the same circumstances, assuming that *P* < 0.05 reflected significant data.

## Results

### Isolation and identification of Gram-negative isolates

Out of the 130 Gram-negative bacterial collections, 117 isolates were phenotypically and biochemically identified and confirmed to be *A. baumannii* (21 isolates, 17.9%), *K. pneumoniae* (40 isolates, 34.2%) and *P. aeruginosa* (56 isolates, 47.9%). For further validation of the *A. baumannii* isolates, multiplex PCR of *recA* and *16–23 S rRNA* genes showed that all the phenotypically identified isolates had the desired genes at 425 and 208 bp, respectively (Additional file 1. Supplementary Fig. 1).

**Fig. 1:**
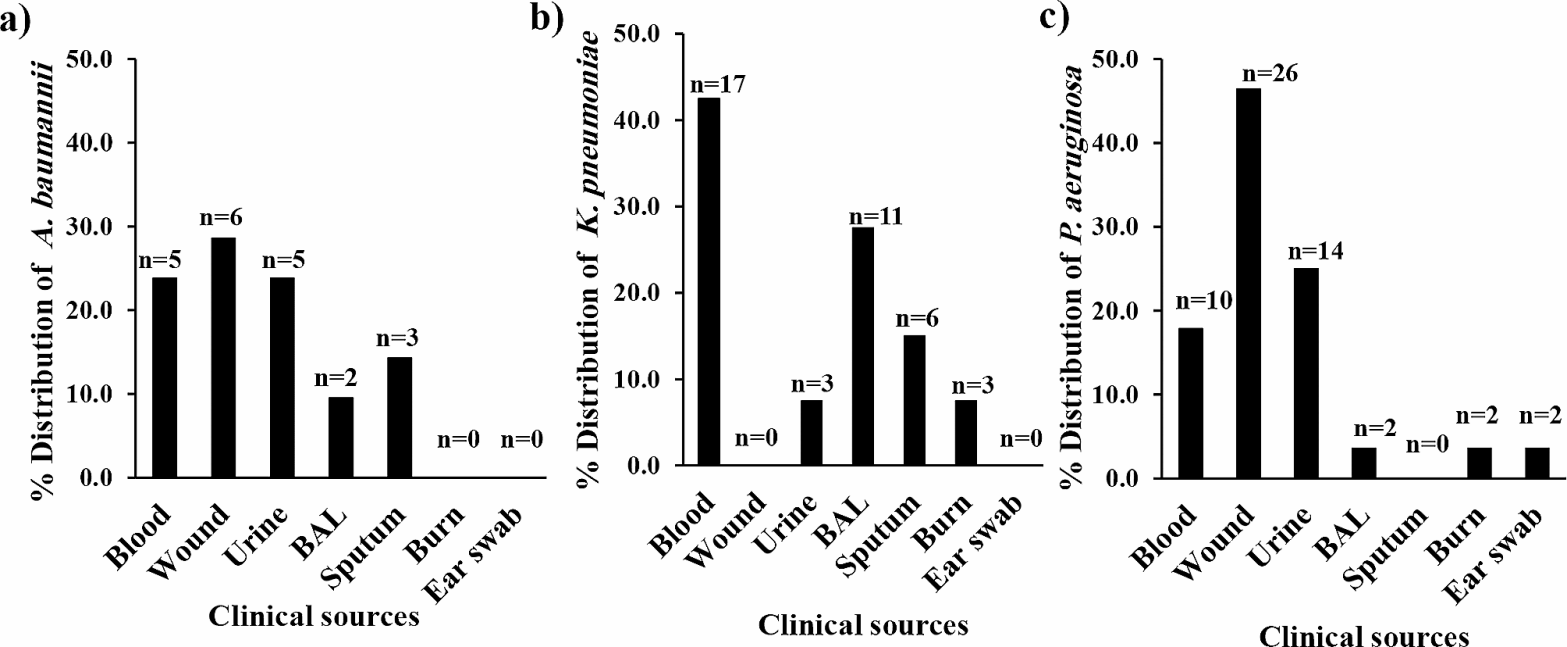
Distribution percentages of (**a**): *A. baumannii*, (**b**): *K. pneumoniae* and (**c**): *P. aeruginosa* among the various clinical sources included in the study. BAL: bronchoalveolar lavage

Isolates were collected from diverse clinical sources, including blood (*n* = 32), wounds (*n* = 32), urine (*n* = 22), BAL fluid (*n* = 15), sputum (*n* = 9), burn tissue (*n* = 5) and ear swabs (*n* = 2; Additional file 1. Supplementary Table 1). Within each genus, the predominant isolates of *A. baumannii* (6 isolates, 28.6%) and *P. aeruginosa* (26 isolates, 46.4%) were from the wound source, while *K. pneumoniae* was isolated in maximum number from blood samples (17 isolates, 42.5%, Fig. 1).

### Antimicrobial sensitivity pattern

**Fig. 2:**
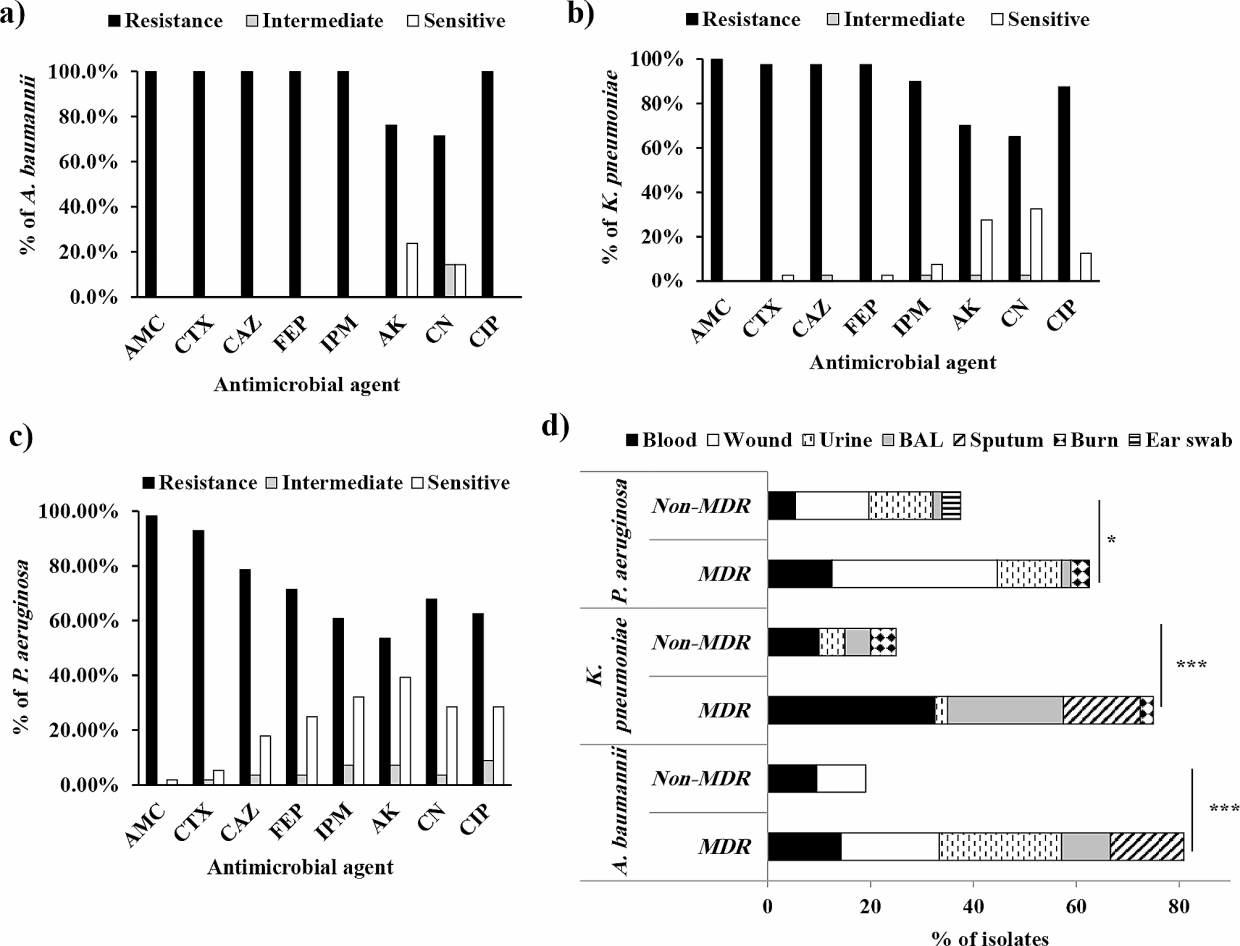
Antibiotic resistance profiles of (**a**): *A. baumannii*, (**b**): *K. pneumonia* and (**c**): *P. aeruginosa* toward different antimicrobial agents, including (**d**) distribution of resistance phenotype categories among different clinical sources. AMC: amoxicillin/clavulanic acid, CTX: cefotaxime, CAZ: ceftazidime, FEP: cefepime, IPM: imipenem, AK: amikacin, CN: gentamicin, CIP: ciprofloxacin, BAL: bronchoalveolar lavage, MDR: multidrug resistance (*, significant, *P* < 0.05)

A disc diffusion test was used to assess the activity of 8 antimicrobial agents belonging to 3 antimicrobial classes against all the collected Gram-negative isolates, and the antibiotic resistance profiles were presented in Additional file 1. Supplementary Table 1. For *A. baumannii*, all the isolates (100%) exhibited resistance to six out of the antibiotics tested, including all the β-lactams and CIP, while showing some susceptibility to AK and CN, at 23.8% and 14.3%, respectively (Fig. 2a). Among *K. pneumonia* isolates, 100% were resistant to AMC, followed by CTX, CAZ, FEP (97.5% each), IPM (90%), CIP (87.5%), AK (70%) and CN (65%, Fig. 2b). Among *P. aeruginosa* isolates (Fig. 2c), the resistance to AMC, CTX, CAZ and FEP was highly prevalent (98.2%, 92.9%, 78.6% and 71.4%, respectively), whereas resistance rates to IPM, AK, CN and CIP were lower than 70%.

Based on CLSI interpretive criteria, isolates that were resistant to three or more antibiotic classes were considered MDR. Notably, MDR strains accounted for 82 (70.1%) of the total Gram-negative isolates. Significantly, the highest proportion of MDR bacteria was *A. baumannii* (81%, *P* = 0.0001), which was mostly observed among the urine samples, while the lowest was *P. aeruginosa* (62.5%, *P* = 0.0463) among the wound samples. The MDR rate among *K. pneumoniae* was 75% (*P* = 0.0029), and most of the isolates were from blood sources (Fig. 2d).

### Assessment of biofilm formation in Gram-negative isolates

**Fig. 3:**
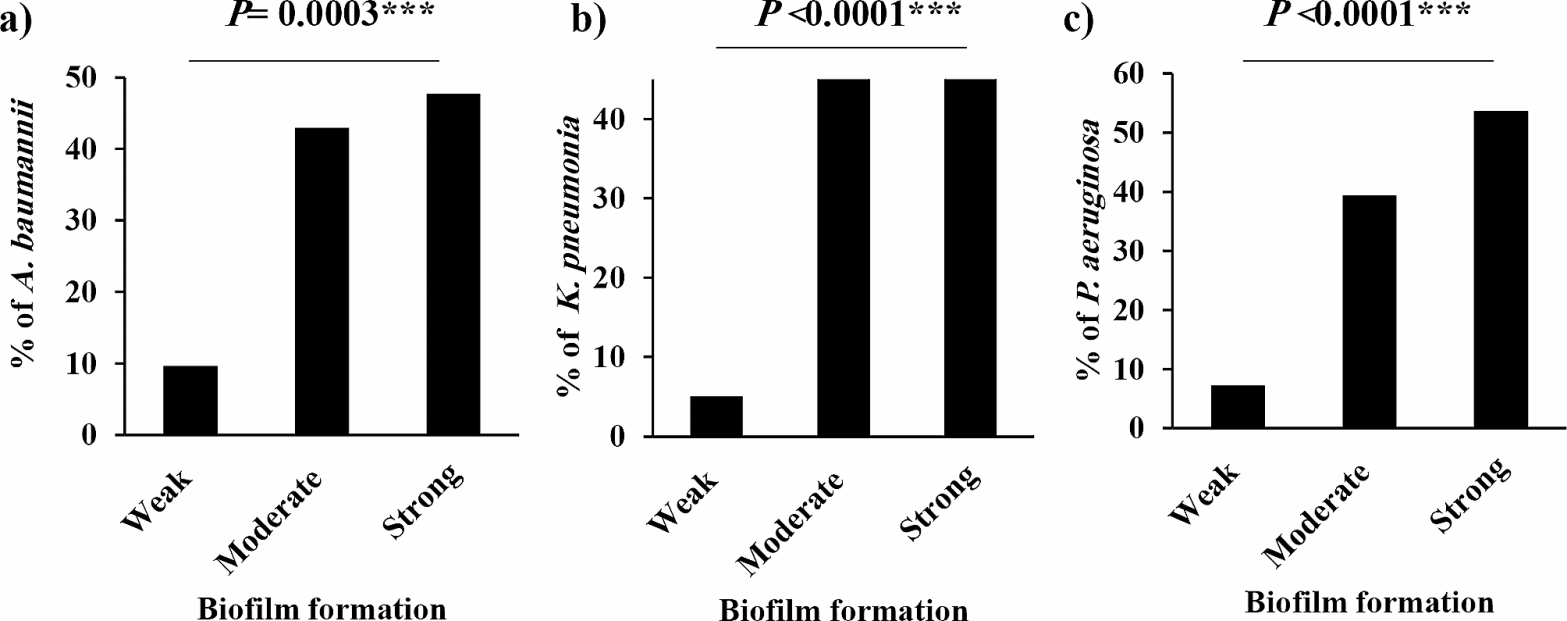
Percentage of isolates categorized as strong, moderate, or weak biofilm producers among the entire group of collected Gram-negative isolates (**a**): *A. baumannii*, (**b**): *K. pneumoniae* and (**c**): *P. aeruginosa* (***, highly significant; *P* < 0.001)

The biofilm-forming capacity of the 117 Gram-negative isolates was evaluated using a microtiter plate assay. All the tested isolates were positive for biofilm formation but had varying adhesion capacities (*P* < 0.001). *A. baumannii* isolates were classified as strong (10 isolates, 47.6%), moderate (9 isolates, 42.9%) or weak (2 isolates, 9.5%) biofilm formers (Fig. 3a). Thirty-eight *K. pneumoniae* isolates produced robust biofilms that were evenly divided into mild and strong producers (47.5% each, Fig. 3b**)**. Among *P. aeruginosa* isolates (Fig. 3c), remarkably high number (*n* = 30, 53.6%) produced denser biofilm biomasses, whereas 4 (7.1%) isolates showed a weak capacity for biofilm production.

### Minimum inhibitory concentrations of the tested vitamins

A series of Gram-negative standard strains, *A. baumannii* (ATCC 19606 and ATCC 17978), *K. pneumoniae* (ATCC 51503) and *P. aeruginosa* PAO1 and PAO14, in addition to some clinical strong biofilm producers of *Acinetobacter* (A5, A20 and A21), *Klebsiella* (K25, K27 and K28), and *Pseudomonas* (P8, P16, P24 and P27), were selected for testing the antimicrobial activity of vitamins D and K1. For vitamin D, the MIC was 1250 µg/ml for all the isolates, except for *A. baumannii* ATCC 17978 and *K. pneumoniae* ATCC 51503 standard strains was 625 µg/ml. On the other hand, vitamin K1 had an MIC value of 2500 µg/ml for the majority of the tested isolates, and 5000 µg/ml was observed for only *A. baumannii* ATCC 17978, A21, *K. pneumoniae* K25 and K28, as well as *P. aeruginosa* PAO1, P8 and P24. Concentrations of vitamins below their MICs (1/2 and 1/4 MICs) were also calculated as described in Table 2.

**Table 2:** **Minimal inhibitory concentrations (MICs) and subinhibitory concentrations (1/2 and 1/4 MICs) of the tested vitamins**

Pharmaceutical supplements	Bacterial isolates	MIC (µg/mL)	1/2 MIC (µg/mL)	1/4 MIC (µg/mL)
**Vitamin D**	***A. baumannii *** **ATCC 17978**	625 ± 0	312.5	156.25
	***A. baumannii *** **ATCC 19606**	1250 ± 0	625	312.5
	**A5**	1250 ± 0	625	312.5
	**A20**	1250 ± 0	625	312.5
	**A21**	1250 ± 0	625	312.5
	***K. pneumoniae *** **ATCC 51503**	625 ± 0	312.5	156.25
	**K25**	1250 ± 0	625	312.5
	**K27**	1250 ± 0	625	312.5
	**K28**	1250 ± 0	625	312.5
	***P. aeruginosa *** **PAO1**	1250 ± 0	625	312.5
	***P. aeruginosa *** **PAO14**	1250 ± 0	625	312.5
	**P8**	1250 ± 0	625	312.5
	**P16**	1250 ± 0	625	312.5
	**P24**	1250 ± 0	625	312.5
	**P27**	1250 ± 0	625	312.5
**Vitamin K1**	***A. baumannii *** **ATCC 17978**	5000 ± 0	2500	1250
	***A. baumannii *** **ATCC 19606**	2500 ± 0	1250	625
	**A5**	2500 ± 0	1250	625
	**A20**	2500 ± 0	1250	625
	**A21**	5000 ± 0	2500	1250
	***K. pneumoniae *** **ATCC 51503**	2500 ± 0	1250	625
	**K25**	5000 ± 0	2500	1250
	**K27**	2500 ± 0	1250	625
	**K28**	5000 ± 0	2500	1250
	***P. aeruginosa *** **PAO1**	5000 ± 0	2500	1250
	***P. aeruginosa *** **PAO14**	2500 ± 0	1250	625
	**P8**	5000 ± 0	2500	1250
	**P16**	2500 ± 0	1250	625
	**P24**	5000 ± 0	2500	1250
	**P27**	2500 ± 0	1250	625

### Effect of vitamins on bacterial growth and viability

The effect of vitamins D and K1, at sub-MICs, on bacterial growth kinetics was studied against three isolates per genus, A5, K28 and P16. As indicated in Fig. ( 4a, b, c), A5, K28 and P16 subjected to 1/2 MIC vitamin D and vitamin K1 grew at a slightly lower rate than did the vitamin-unexposed isolates. However, no significant difference in the final OD600 nm was noted between cultures with untreated broth and their counterparts with broth containing 1/2 MIC of the tested vitamins, indicating that the presence of vitamin D or vitamin K1 at sub-MICs did not hamper the bacterial growth over 24 h.

**Fig. 4:**
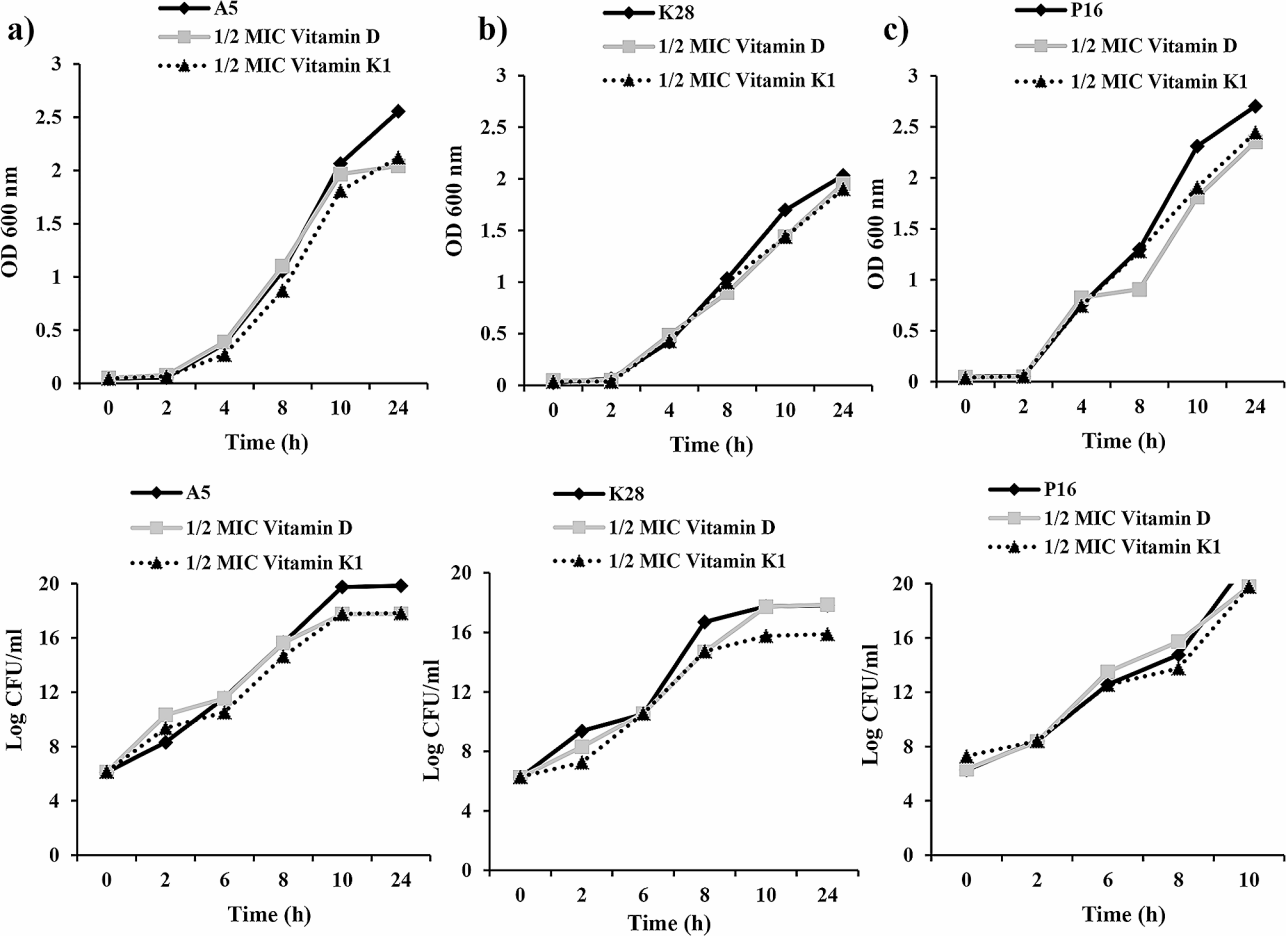
Growth curve (OD 600 nm) and Viable counting (log CFU/ml) of (**a**): *A. baumannii* A5, (**b**): *K. pneumoniae* K28 and (**c**): *P. aeruginosa* P16 in absence and presence of 1/2 MIC of vitamin D and vitamin K1. Data represent the mean of three independent experiments

To complement the previous growth curve data, viable bacteria were also counted. Again, no considerable reduction in log CFU/mL was observed for either pathogen after 1/2 MIC exposure to vitamin D or vitamin K1 **(**Fig. 4d, e, f**)**.

### Effects of vitamin D and vitamin K1 on bacterial attachment

**Fig. 5:**
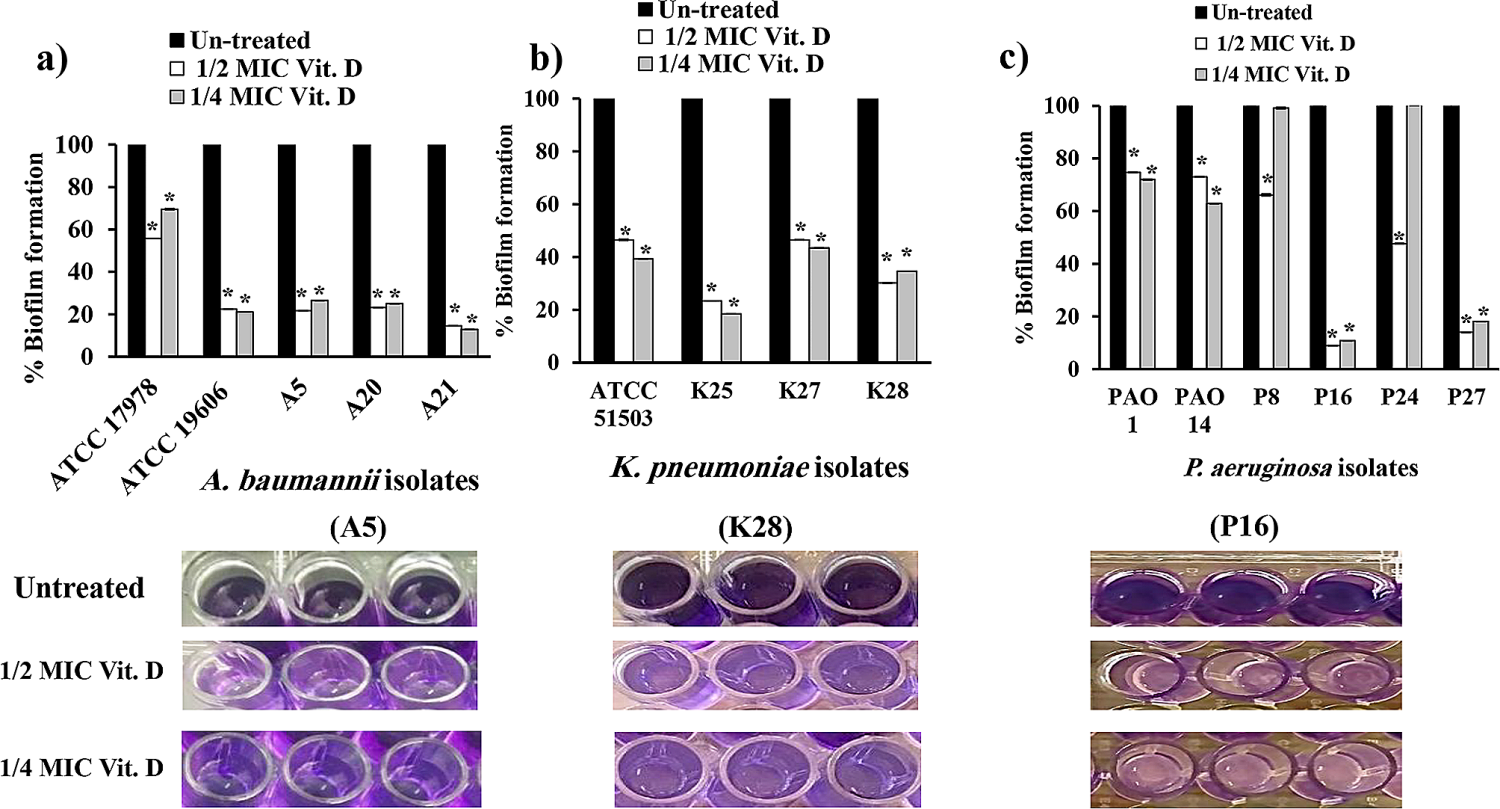
Effect of vitamin D at sub-MICs (1/2 and 1/4 MICs) on the initial adherence and biofilm configuration in (**a**): *A. baumannii* (ATCC 19606 and ATCC 17978 standard strains and A5, A20 and A21 clinical isolates); (**b**): *K. pneumoniae* (ATCC 51503 standard strain and K25, K27 and K28 clinical isolates); and (**c**): *P. aeruginosa* (PAO1 and PAO14 standard strains and P8, P16, P24 and P27 clinical isolates) in relation to untreated cultures. Error bars represent the standard deviation of three independent repetitions (*, significant, *P* < 0.05)

The CV assay was employed to assess the antiadherence and subsequent biofilm formation capacity of vitamin D and vitamin K1 at their respective sub-MICs against the tested standard and the previously selected strong biofilm-producing isolates. Compared to the dense biofilm formed by untreated cultures, vitamin D significantly reduced biofilm formation by 44–85% at 1/2 MIC and by 30–87% at 1/4 MIC in *A. baumannii* (Fig. 5a). In *K. pneumoniae*, vitamin D revealed a significant decrease at 1/2 and 1/4 MIC by 53–77% and 57–82%, respectively (Fig. 5b). Similarly, all tested isolates of *P. aeruginosa* treated with sub-MICs of vitamin D significantly decreased the biofilm architecture in the range of 25–91%, except for only two isolates; P8 and P24 (at 1/4 MICs) showed poor biofilm eradication (Fig. 5c, *P* < 0.0001).

**Fig. 6:**
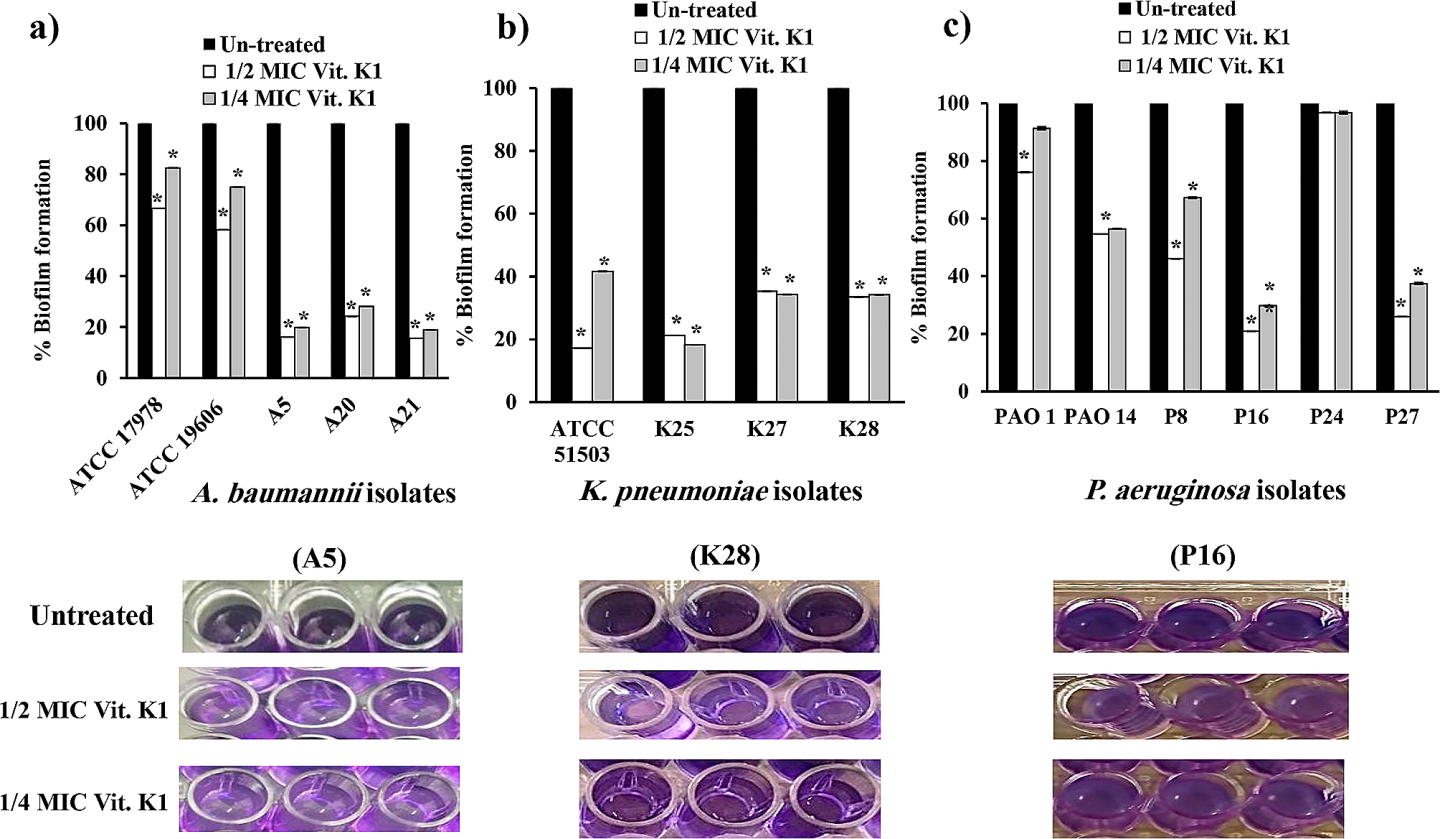
Effect of 1/2 and 1/4 MICs of vitamin K1 on initial adherence and biofilm configuration in (**a**): *A. baumannii* (ATCC 19606 and ATCC 17978 standard strains and A5, A20 and A21 clinical isolates); (**b**): *K. pneumoniae* (ATCC 51503 standard strain and K25, K27 and K28 clinical isolates); and (**c**): *P. aeruginosa* (PAO1 and PAO14 standard strains and P8, P16, P24 and P27 clinical isolates) in relation to untreated cultures. Error bars represent the standard deviation of three independent repetitions (*, significant, *P* < 0.05)

Compared to the untreated cells, vitamin K1 significantly (*P* < 0.0001) suppressed the initial stage of *A. baumannii* biofilm formation in a concentration-dependent manner by 33–84% and 17–81% at 1/2 and 1/4 MIC, respectively (Fig. 6a). It also reduced biofilm formation by 65–83% at 1/2 MIC and by 58–82% at 1/4 MIC in *K. pneumoniae* (Fig. 6b). The lowest activity of vitamin K1 was observed in *P. aeruginosa*, as it diminished biofilm production at 1/2 and 1/4 MIC by 3–79% and 3–70%, respectively. The poorest performance of vitamin K1 on initial adherence and biofilm assembly was manifested in PAO1 (at 1/4 MIC) and P24 (at 1/2 and 1/4 MICs, Fig. 6c).

#### Impact of vitamin D and vitamin K1 on mature biofilm inhibition

**Fig. 7:**
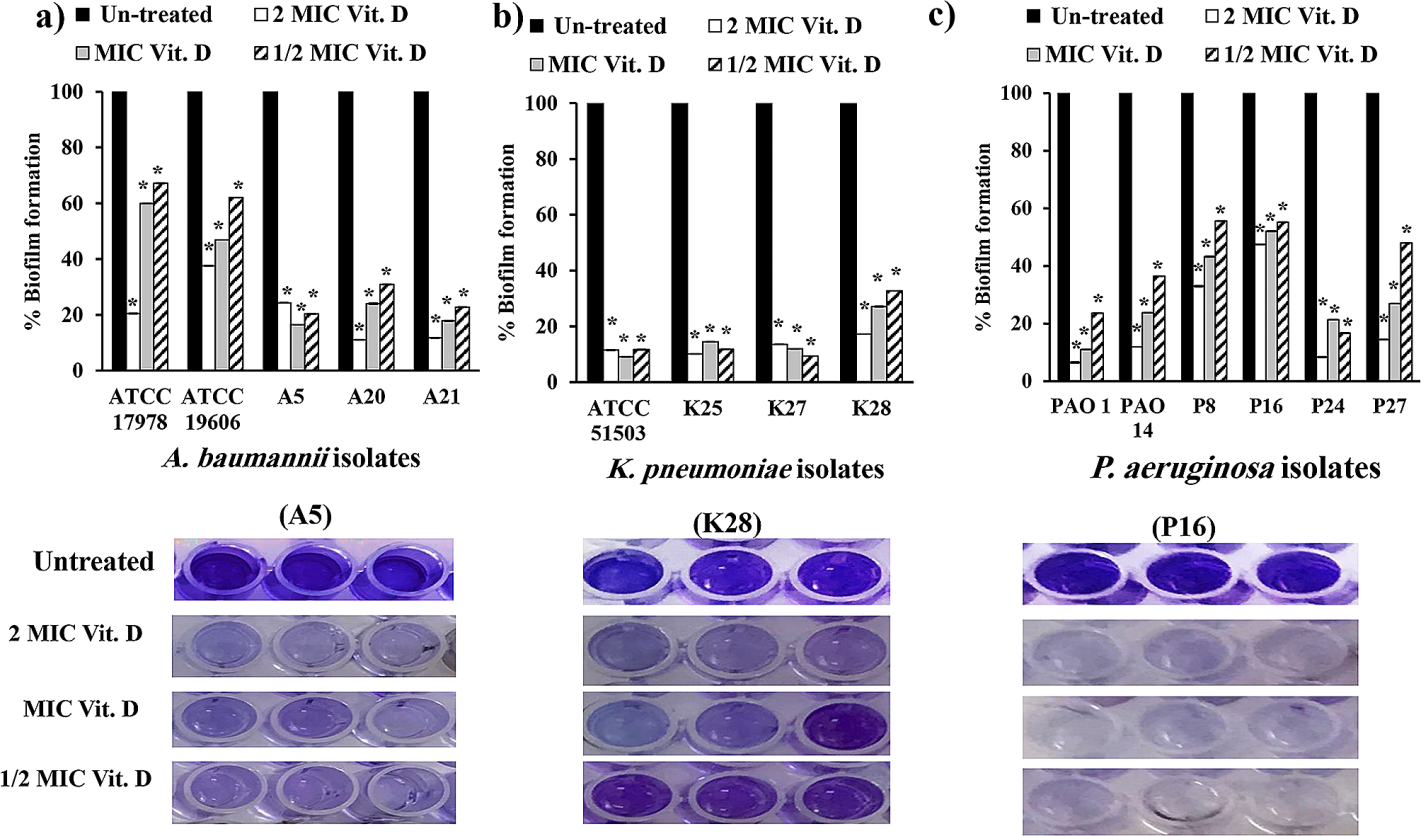
Effect of different concentrations (0.5×, 1× and 2× MIC) of vitamin D on the mature biofilm architecture of (**a**): *A. baumannii* (ATCC 19606 and ATCC 17978 standard strains and A5, A20 and A21 clinical isolates); (**b**): *K. pneumoniae* (ATCC 51503 standard strain and K25, K27 and K28 clinical isolates); and (**c**): *P. aeruginosa* (PAO1 and PAO14 standard strains and P8, P16, P24 and P27 clinical isolates) compared with unchallenged cells. Error bars represent the standard deviation of three independent experiments (*, significant, *P* < 0.05)

The influence of different concentrations of vitamins D and K1 on the mature biofilm formed by the selected standard and clinical Gram-negative bacilli was compared to that on the untreated mature biofilm using microtiter plates. Vitamin D at different doses significantly reduced mature biofilm production, exhibiting notable activity on *K. pneumoniae* isolates, with 67–91%, 73–91% and 83–90% reductions at 0.5×, 1× and 2× MIC, respectively (Fig. 7b). Likewise, vitamin D exhibited significant reduction in *A. baumannii* (Fig. 7a) and *P. aeruginosa* (Fig. 7c) by 33–83% and 44–83%, respectively, at 0.5× MIC and by 40–83% and 48–89%, respectively, at 1× MIC, in addition to 62–89% and 53–94%, respectively, at 2× MIC.

**Fig. 8:**
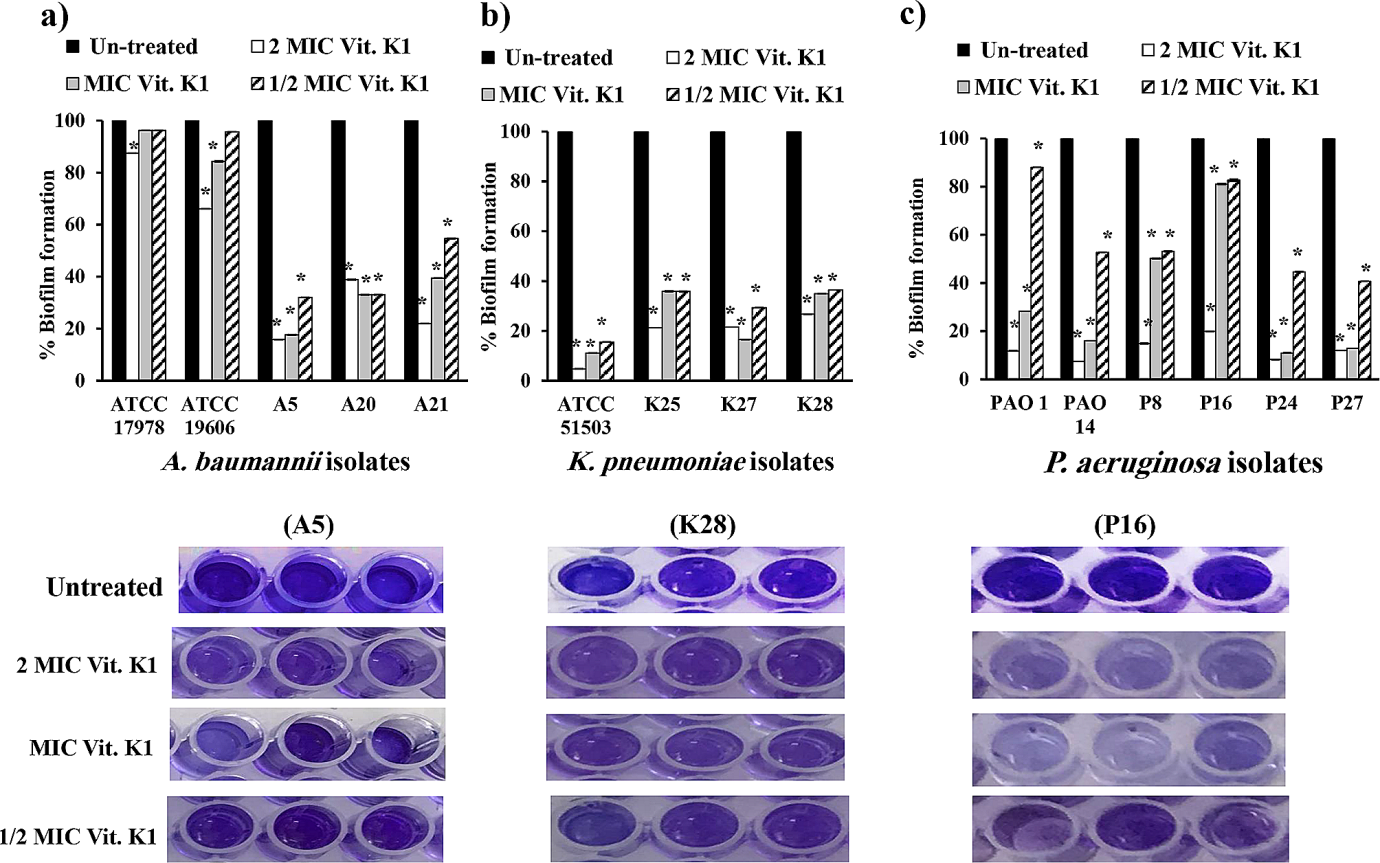
Effect of different concentrations (0.5×, 1× and 2× MIC) of vitamin K1 on the mature biofilm architecture of (**a**): *A. baumannii* (ATCC 19606 and ATCC 17978 standard strains and A5, A20 and A21 clinical isolates); (**b**): *K. pneumoniae* (ATCC 51503 standard strain and K25, K27 and K28 clinical isolates); and (**c**): *P. aeruginosa* (PAO1 and PAO14 standard strains and P8, P16, P24 and P27 clinical isolates) compared with unchallenged cells. Error bars represent the standard deviation of three independent experiments (*, significant, *P* < 0.05)

The degradation effect of vitamin K1 was most pronounced in *K. pneumonia*, ranging from 64 to 84% at 0.5× MIC to 64–89% at 1× MIC and from 73 to 95% at 2× MIC (Fig. 8b). Mature *A. baumannii* biofilms treated with vitamin K1 were substantially eliminated, ranging from 45% at 0.5× MIC to 84% at 2× MIC (Fig. 8a). Additionally, 0.5×, 1× and 2× the MIC of vitamin K1 decreased *P. aeruginosa* biofilms by 17–59%, 19–89% and 80–93%, respectively (Fig. 8c).

### Molecular identification of biofilm-regulatory and housekeeping genes

#### Polymerase chain reaction (PCR)

PCR detection of the genes responsible for biofilm formation, *cusD*, *bssS* and *pelA*, revealed that these genes were carried by the representative isolates A5, K28 and P16, respectively (Additional file 1. Supplementary Fig. 2a). Furthermore, the presence of the standard genes *rpoB* in *A. baumannii* (A5), * ropD* in *K. pneumoniae* (K28) and * ropD* in *P. aeruginosa* (P16) was confirmed via PCR. The results showed that each genus completely harboured its reference-specialized gene (Additional file 1. Supplementary Fig. 2b).

#### Gene expression by qPCR

**Fig. 9:**
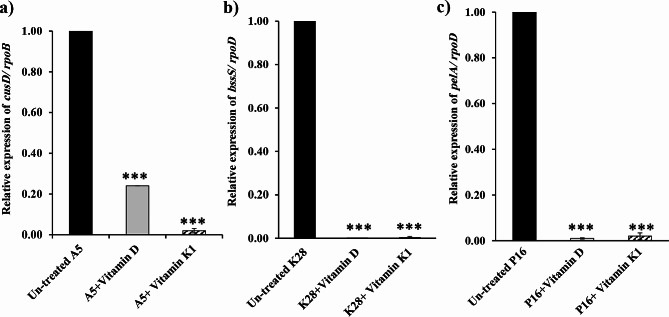
Relative expression of biofilm-regulatory genes (**a**): *cusD/rpoB* in *A. baumannii* A5, (**ba**) *bssS/ ropD* in *K. pneumoniae* K28 and (**c**) *pelA/ ropD* in *P. aeruginosa* P16 treated with vitamin D and K1 (at their 0.5× MIC) compared with that in untreated isolates. Error bars represent the standard deviation of three independent results (***, highly significant, *P* < 0.0001)

The relative expression of biofilm-forming genes in cultures treated with 1/2 MIC of vitamin D or vitamin K1 compared to that in untreated controls was displayed in Fig. 9. The Ct values of each tested gene (*cusD*, *bssS* and *pelA*) were determined, and their relative amounts were then normalized to those of the reference genes *rpoB*, * ropD* and * ropD* in the same sample. In *A. baumannii* A5, both vitamin D and vitamin K1 significantly reduced *cusD* gene expression by 76% and 98%, respectively (Fig. 9a). Similarly, upon treatment of *K. pneumoniae* K28 with vitamins D or K1, the expression of *bssS* was significantly reduced by 100% and 99.7%, respectively (Fig. 9b). In addition, exposure of *P. aeruginosa* P16 to vitamins D and K1 caused significant downregulation of *pelA*, by 99% and 98%, respectively (Fig. 9c).

## Discussion

Antibiotic resistance is a concerning and formidable phenomenon because, in addition to its contribution to increasing the rate of morbidity and mortality, it is also associated with biofilm formation in numerous pathogens, especially Gram-negative organisms [41]. This study focused on three of the most common resistant strains encountered in hospitals, namely, *A. baumannii*, *K. pneumoniae* and *P. aeruginosa*. A total of 117 Gram-negative isolates were collected from different clinical sources; *P. aeruginosa* was the most prevalent pathogen, accounting for 47.9%, followed by *K. pneumoniae* (34.2%). The lowest incidence was for *A. baumannii* (17.9%), which is similar to the results recorded by Sundaram and coauthors (21%) [42] and Rabina and colleagues (20%) [43]. The maximum prevalence of *K. pneumoniae* was observed in blood cultures (42.5%), as previously detected [44–46]. Most of the *A. baumannii* and *P. aeruginosa* strains were isolated from wound samples (28.6% and 46.4%, respectively), which is consistent with the finding of previous studies by Ruh et al. and Farajzadeh et al. [45, 47]. Notably, only two isolates of *P. aeruginosa* were purified from ear swabs, as *P. aeruginosa* is an opportunistic pathogen that can enter through a pierced ear, causing severe middle ear infections [48, 49] (Fig. 1, Additional file 1. Supplementary Table 1).

The antibiotic resistance pattern demonstrated a concerning level of resistance across all the collected strains toward almost all the antimicrobial agents tested (Fig. 2, Additional file 1. Supplementary Table 1). The resistance to the tested β-lactam antibiotics was excessively high, ranging from more than 60% against IPM in *P. aeruginosa* to complete resistance (100%) towards AMC in *A. baumannii* and *K. pneumoniae*. This could be due to the usual and widespread usage of β-lactams without proper consideration of disease severity or following microbiological diagnostic procedures [50]. Similar findings have been reported in many previous studies [51–54]. Additionally, high resistance to aminoglycosides and CIP was observed against all the tested isolates, which is in accordance with the findings of other studies [51, 55–57].

Additionally, most of the isolates were MDR (70.1%), with incidences of 81, 75 and 62.5% for *A. baumannii, K. pneumoniae* and *P. aeruginosa*, respectively (Fig. 2d). A similar MDR prevalence in *A. baumannii* and *K. pneumoniae* has been previously reported [58]. The ability of microbes to utilize various resistance mechanisms to evade the effects of antibiotics and to overcome the emergence of high resistance to critical antimicrobial classes is a great challenge, as described by the WHO. This disaster also worsened because of prominent nonsusceptibility to carbapenem (imipenem, Fig. 2), which is considered the last resort antimicrobial agent reserved for fighting serious bacterial infections [59]. Therefore, the increase in MDR phenotypes and treatment of patients infected with these bacteria pose significant challenges [60]. Notably, the MDR incidence was significantly greater for *A. baumannii* isolated from urine specimens (29.4%) than for those isolated from other sources, which indicated that the treatment of urinary tract infections caused by this pathogen is a concerning issue. Furthermore, MDR *P. aeruginosa* was detected in 58% of the wound samples, suggesting that there was too much post-surgical antibiotic usage and hospital readmission.

Biofilm formation is an intrinsic weapon for pathogenic and opportunistic isolates in various clinical settings. In this study, nearly all the isolates (93.2%, Fig. 3) were moderate or strong biofilm producers, and this proportion was much greater than that reported in other studies [61] (62.73%) [62], (71.8%) and [63] (87.5%). Consequently, it is crucial to diagnose and treat infections as early as possible prior to biofilm development, as this approach could enhance the response to antimicrobial therapy [64, 65]. Even poor biofilm-producing phenotypes may still be risky during polymicrobial infections where they can be integrated into preexisting biofilms or provide a synergistic effect with other robust biofilm formers [66].

The majority of strong biofilm producers were extracted from wounds (32.2%) more than from blood (25.4%) or other clinical sources, supporting the fact that that surgical wounds, skin lesions or any abrasion in mammalian tissues due to implanted medical devices favours the formation and evolution of bacterial biofilms [67, 68]. This result was consistent with those reported by Leshem et al. and Piechota et al. [69, 70]. MDR isolates had a more robust ability to form biofilms than did their counterparts, similar to the finding of Dumaru and colleagues [68]. Biofilm architecture plays a crucial role in fortifying bacteria within a biofilm matrix against classical and even broad-spectrum antibiotics, allowing them to cause notorious and devastating diseases in healthy and immunocompromised individuals. Also, many researchers have shown a link between biofilm creation ability and resistance to certain antimicrobial agents [71–73]. The emergence of small colony variants (SCVs), which remain viable inside the bacterial population at a slow growth rate, has been reported to be associated with special biofilm formation abilities and MDR [74]. A bacterial switch to the SCV phenotype provides a significant colonization advantage, causing recurrent and aggressive infection. *P. aeruginosa* has been reported to produce SCV with an overproduction of EPS and a strong ability to form biofilms [75, 76]. Silva and coauthors reported the development of colistin-resistant biofilms in *K. pneumoniae* with SCV characteristics [77].

The capability of *A. baumannii*, *K. pneumoniae* and *P. aeruginosa* superbugs to develop biofilms provides insight into how these organisms escape antimicrobial therapies and cause life-threatening infections [11, 78]. Therefore, there is a need for effective approaches to tackle AMR. Hence, the interruption of Gram-negative biofilms by vitamin D and vitamin K1 at different concentrations was studied against some strong biofilm-producing isolates. Vitamins D and K1 possessed antimicrobial activity at concentrations ranging from 625 to 1250 µg/ml and 2500–5000 µg/ml, respectively (Table 2). The antimicrobial effects of vitamin D3 on both Gram-positive bacteria, such as *S. aureus* and *S. pyogenes*, and Gram-negative bacteria, including *K. pneumoniae* and *E. coli* were estimated [79]. The reported antimicrobial activity of both vitamins D and K1 was associated with lipid solubility and alterations in membrane permeability [80]. In the study of Andrade and colleagues, vitamin K3 was shown to possess antimicrobial activity, with an MIC of 64 µg/ml against *P. aeruginosa* 03, and to potentiate the antimicrobial activity of aminoglycosides [80].

The antimicrobial activity of both vitamins at the 1/2 MIC was monitored over a 24 h period by measuring the OD and CFU/ml of treated *A. baumannii* A5, *K. pneumoniae* K28 and *P. aeruginosa* P16 and comparing the obtained data with those of untreated cells cultivated under the same conditions **(**Fig. 4**)**. Both the control and vitamin-challenged cultures grew exponentially, indicated by the lack of significant differences in the typical growth curves. Furthermore, vitamin D and vitamin K1 were not shown to have an effect on the CFU/ml of A5, K28 or P16 at 1/2 MIC.

The antibiofilm effects of vitamin D and vitamin K1 were phenotypically and genotypically assessed using concentrations of 1/2 MIC, or less (1/4 MIC), of both vitamins. Vitamins D and K1, below their MICs, significantly inhibited bacterial adhesion at 1/2 MIC more than 1/4 MIC, particularly among *K. pneumoniae* isolates (Figs. 5 and 6). Even after the biofilm of standard and clinical *A. baumanni*, *K. pneumoniae* and *P. aeruginosa* isolates was maturated after 24 h; vitamin D and K1 could effectively disrupt the biofilms and significantly reduce the bacterial biofilm density at concentrations of 0.5×, 1× and 2× the MIC (Figs. 7 and 8). This biofilm-hindering ability of both vitamins may be due to many factors that are responsible for forming and supporting the bacterial biofilm architecture, such as affecting EPS matrix production, bacterial motility, intra or intercellular communication or any other environmental conditions [81, 82]. Previous studies have evaluated different vitamin K types, including vitamins K1, K2 and K3. Soltani et al. elucidated the anti-quorum sensing and biofilm formation effects of vitamin K1, particularly through the inhibition of pqs pathway in *P. aeruginosa* [83]. Supplementation with vitamin K3 decreased susceptibility to cell wall inhibitors and other antimicrobial agents, triggered EPS synthesis and subsequently increased the biofilm biomass content of *S. aureus* SCVs more than that of the wild-type *S. aureus* strain [84]. Nonetheless, vitamin K3 has good antimicrobial and antibiofilm effects [85].

On the other hand, sub-MICs of some antimicrobial agents, such as aminoglycosides, increase biofilm formation by *P. aeruginosa* and *E. coli* [86, 87]. In addition, carbenicillin, tetracycline and colistin enhanced biofilm formation in *E. coli* and *A. baumannii* [88, 89]. Triclosan, a common bactericidal agent used in cosmetics, has short-term antibiofilm activity, as detected by Ricart and coauthors [90], and is typically tolerated by the bacterial community in the biofilm matrix within a few days [91]. Carbapenems (imipenem, meropenem and doripenem) altered the biofilm morphology without affecting the viability of treated biofilms compared to untreated cells in *K. pneumoniae* [92] and induced the expression of alginate genes, leading to increased biofilm density in *P. aeruginosa* [93]. Similarly, in Gram-positive bacteria, some β-lactams such as ampicillin, amoxicillin and cloxacillin, hinder the release of extracellular DNA which leads to increased biofilm volume in *S. aureus* [94] and *E. faecalis* [95]. Besides, biofilm communities resist the destructive effect of conventional antibiotics because of the constant change in their EPS matrix [96]. Therefore, antibiotics enhance biofilm evolution and resistance, leading to the environmental dissemination of resistant phenotypes and genotypes [97].

Previous studies have demonstrated the in vivo activity of vitamin D and its importance for normal intestinal homeostasis, with a reported effect on bacterial colonization [98]. Moreover, vitamin D stimulates the production of antimicrobial peptides that maintain the barrier function of the gut microbiota and airway function [99]. Increasing the vitamin D concentration to more than 30–40 ng mL^− 1^ in human serum, reduces the risk of biofilm-associated caries [100]. Additionally, vitamin D is a promising adjuvant therapy for managing biofilm-related bacterial infections due to its immunomodulatory activity, as it can induce the secretion of cathelicidin, an antimicrobial peptide, while decreasing the secretion of inflammatory cytokines [101]. Furthermore, vitamin K has a beneficial impact on the intestinal microbiota by preventing or treating intestinal disorders associated with microbial imbalances [102], including inflammatory bowel disorders [103].

To confirm the antibiofilm effect of both D and K1, the transcriptional response of the biofilm-regulatory genes *cusD*, *bssS* and *pelA* was assessed against the A5, K28 and P16 tested isolates, respectively. Compared with those in the corresponding nontreated cells, the expression levels of *cusD* in A5, *bssS* in K28 and *pelA* in P16 were markedly downregulated by 76–100% under both vitamin treatment conditions (Fig. 9). Specifically, vitamin D and vitamin K1 significantly reduced *cusD* gene expression in *A. baumannii* A5 by 76% and 98%, respectively (Fig. 9a). In *A. baumannii*, *cusD* is involved in cellular motility, surface adhesion and further biofilm formation [104]. The overexpression of csuA/ABCDE is attributed to *A. baumannii* adherence, increased mushroom-like biofilm formation, and decreased bacterial motility [105]. Therefore, the suppression of *cusD* gene could be associated with a reduction in bacterial colonization of surfaces and the subsequent inability to germinate biofilms.

Treatment of *K. pneumoniae* K28 with vitamins D and K1 significantly reduced the expression of *bssS* by 100% and 99.7%, respectively (Fig. 9b). Additionally, *P. aeruginosa* P16 treated with vitamins D and K1 exhibited significant downregulation of *pelA* expression by 99% and 98%, respectively (Fig. 9c). Both *bssS* and *pelA* are biofilm-associated small proteins that have a substantial functions in the synthesis and transport of EPS across *K. pneumoniae* and *P. aeruginosa* cells, respectively, thereby regulating biofilm maturation [106, 107]. Hence, suppression of *pelA* gene could be associated with the lack of pel polysaccharide-dependent biofilm formation, which is the primary exopolysaccharide biofilm matrix in *P. aeruginosa* [108]. Similarly, the expression of biofilm-related genes in MRSA (*icaA* and *icaR*) and *P. aeruginosa* (*lecA* and *pelA*) was downregulated by vitamin K and vitamin C, respectively [15, 109].

Therefore, this study provides prospective and promising potential for the usage of frequently consumed pharmaceutical supplements (vitamins D and K1) in the eradication of difficult-to-treat biofilm-triggered infections. However, potential antibiofilm endeavour of vitamins D and K1 were detected using representative *A. baumannii, K. pneumoniae* and *P. aeruginosa* isolates, and further research is needed to confirm these findings in other Gram-negative isolates and to identify other Gram-positive and MDR organisms. Additionally, in vivo analysis will provide deeper insights into the clinical applications of the obtained effects.

## Conclusion

In summary, this work focused on studying the most common Gram-negative bacilli in hospital settings: *A. baumannii*, *K. pneumoniae* and *P. aeruginosa.* The significant increase in resistance, including MDR, and the increase in the prevalence of biofilm-forming isolates reflect an emerging threat posed by these superbugs in our country. Our results revealed that the two vitamins D and K1 exerted antimicrobial effects on the tested isolates at their pharmaceutical dosage. Moreover, they hindered biofilm attachment and disrupted its mature structure in the tested strains. This inhibitory effect was confirmed by the significant decrease in the expression of the genus-specific biofilm-encoding genes *cusD*, *bssS* and *pelA*. This antibiofilm property of both vitamins holds significant value in clinical therapies against the tested isolates. Coadministration of antibacterial agents with these vitamins could reduce or reverse resistance. However, further research is required to elucidate the in vivo activity of these vitamins.

### Electronic supplementary material

Below is the link to the electronic supplementary material.

Supplementary Material 1

Supplementary Material 2

Supplementary Material 3

## Data Availability

All the data generated and analysed during the study are included in this manuscript and its supplementary document. The raw datasets are available from the corresponding author upon request.

## Abbreviations

*A. baumannii*: *Acinetobacter baumannii*
*K. pneumoniae*: *Klebsiella pneumoniae*
*P. aeruginosa*: *Pseudomonas aeruginosa*
*S. aureus*: *Staphylococcus aureus*
AMR: Antimicrobial resistance
MDR: Multidrug resistance
EPS: Extracellular polymeric substances
BAL: Bronchoalveolar lavage
IMVic: indole, methyl red, Voges-Proskauer, and citrate utilization
PCR: Polymerase chain reaction
*ITS*: Intergenic spacer
CLSI: Clinical Laboratory Standard Institute
AMC: Amoxicillin/clavulanic acid
CTX: Cefotaxime
CAZ: Ceftazidime
FEP: Cefepime
IPM: Imipenem
AK: Amikacin
CN: Gentamicin
CIP: Ciprofloxacin
TSB: Tryptic soy broth
MHB: Muller-Hinton broth
ODc: Cut-off OD
CV: Crystal violet
MIC: Minimum inhibitory concentration
LB broth: Luria–Bertani broth
CFUs: Colony-forming units
RT-PCR: Real-time polymerase chain reaction
SCVs: Small colony variants
