# Supplementary material for: Vitamin D and vitamin K1 as novel inhibitors of biofilm in Gram-negative bacteria
Source: BMC Microbiol. 2024 May 18;24:173. doi: 10.1186/s12866-024-03293-6 (PMC11102130; doi:10.1186/s12866-024-03293-6)
Supplement: Supplementary file 1 — Supplementary Material 1 [file 12866_2024_3293_MOESM1_ESM.pdf]

Vitamin D and Vitamin K1 as novel inhibitors of biofilm in Gram-negative bacteria

Lekaa L. Lutfi[1,2], Mona I. Shaaban[1]* and Soha Lotfy Elshaer[1]

1 Department of Microbiology and Immunology, Faculty of Pharmacy, Mansoura University, Mansoura 35516, Egypt

2 Department of Microbiology and Immunology, Faculty of Pharmacy, Horus University-Egypt, New Damietta, Egypt

*Correspondence: Mona I. Shaaban

Mona_ibrahem@mans.edu.eg

Keywords

Gram-negative bacteria, *A. baumannii*, *K. pneumoniae*, *P. aeruginosa*, antimicrobial resistance, biofilm formation, vitamin D, vitamin K1

Supplementary Table1: Antimicrobial susceptibility profile and clinical sources of all tested Gram-negative isolates

Isolate code	Clinical source	β-Lactams					Aminoglycosides		Quinolones
		Penicillin	Cephalosporins			Carbapenems			
		AMC	CTX	CAZ	FEP	IPM	AK	CN	CIP
**A1**	Sputum	R	R	R	R	R	R	R	R
**A2**	Urine	R	R	R	R	R	S	R	R
**A3**	Urine	R	R	R	R	R	R	R	R
**A4**	Wound	R	R	R	R	R	R	R	R
**A5**	Urine	R	R	R	R	R	R	R	R
**A6**	Wound	R	R	R	R	R	S	S	R
**A7**	Blood	R	R	R	R	R	S	S	R
**A8**	Blood	R	R	R	R	R	R	R	R
**A9**	Wound	R	R	R	R	R	R	R	R
**A10**	BAL	R	R	R	R	R	R	R	R
**A11**	BAL	R	R	R	R	R	R	R	R
**A12**	Urine	R	R	R	R	R	R	I	R
**A13**	Wound	R	R	R	R	R	R	R	R
**A14**	Urine	R	R	R	R	R	R	R	R
**A15**	Wound	R	R	R	R	R	R	I	R
**A16**	Blood	R	R	R	R	R	R	R	R
**A17**	Blood	R	R	R	R	R	R	R	R
**A18**	Sputum	R	R	R	R	R	R	R	R
**A19**	Sputum	R	R	R	R	R	R	R	R
**A20**	Wound	R	R	R	R	R	S	I	R
**A21**	Blood	R	R	R	R	R	S	S	R

A: *Acinetobacter baumanii*, **BAL**: Broncho-alveolar lavage, **AMC:** Amoxicillin/clavulanic acid, **CTX:** Cefotaxime, **CAZ:** Ceftazidime, **FEP:** Cefepime, **IPM:** Imipenem, **AK:** Amikacin, **CN:** Gentamicin, **CIP:** Ciprofloxacin, **R:** Resistant, **I:** Intermediate, **S:** Sensitive

Isolate code	Clinical source	β-Lactams					Aminoglycosides		Quinolones
		Penicillin	Cephalosporins			Carbapenems			
		AMC	CTX	CAZ	FEP	IPM	AK	CN	CIP
**K1**	Blood	R	R	R	R	R	S	I	R
**K2**	Blood	R	R	R	R	R	R	S	R
**K3**	Blood	R	R	R	R	R	S	S	R
**K4**	Blood	R	R	R	R	R	R	R	R
**K5**	Blood	R	R	R	R	R	R	R	R
**K6**	BAL	R	R	R	R	R	R	R	R
**K7**	BAL	R	R	R	R	R	R	R	R
**K8**	Blood	R	R	R	R	R	R	R	R
**K9**	BAL	R	R	R	R	R	R	R	R
**K10**	BAL	R	R	R	R	R	R	R	R
**K11**	Sputum	R	R	R	R	R	R	R	R
**K12**	Sputum	R	R	R	R	R	R	S	R
**K13**	Blood	R	R	R	R	R	R	R	R
**K14**	Blood	R	R	R	R	R	R	R	R
**K15**	Blood	R	R	R	R	R	R	R	R
**K16**	Blood	R	R	R	R	R	R	R	R
**K17**	BAL	R	R	R	R	R	R	R	R
**K18**	Blood	R	R	R	R	R	R	R	R
**K19**	Blood	R	R	R	R	R	R	R	R
**K20**	Blood	R	R	R	R	R	R	R	R
**K21**	Sputum	R	R	R	R	R	S	R	R
**K22**	Blood	R	R	R	R	R	R	R	R
**K23**	Burn	R	R	R	R	S	S	R	S
**K24**	Urine	R	R	R	R	R	R	S	R
**K25**	Burn	R	R	R	R	R	R	R	R
**K26**	Burn	R	S	I	S	I	S	S	S
**K27**	Urine	R	R	R	R	S	S	R	S
**K28**	Urine	R	R	R	R	R	S	S	S
**K29**	Blood	R	R	R	R	R	S	S	R
**K30**	Blood	R	R	R	R	R	S	S	R
**K31**	Blood	R	R	R	R	R	S	R	R
**K32**	BAL	R	R	R	R	R	R	S	R
**K33**	BAL	R	R	R	R	R	S	S	R
**K34**	BAL	R	R	R	R	R	R	R	R
**K35**	BAL	R	R	R	R	R	R	R	R
**K36**	BAL	R	R	R	R	R	R	R	R
**K37**	BAL	R	R	R	R	S	I	S	S
**K38**	Sputum	R	R	R	R	R	R	R	R
**K39**	Sputum	R	R	R	R	R	R	S	R
**K40**	Sputum	R	R	R	R	R	R	S	R

K: *Klebsiella pneumonia*, **BAL**: Broncho-alveolar lavage, **AMC:** Amoxicillin/clavulanic acid, **CTX:** Cefotaxime, **CAZ:** Ceftazidime, **FEP:** Cefepime, **IPM:** Imipenem, **AK:** Amikacin, **CN:** Gentamicin, **CIP:** Ciprofloxacin, **R:** Resistant, **I:** Intermediate, **S:** Sensitive

Isolate code	Clinical source	β-Lactams					Aminoglycosides		Quinolones
		Penicillin	Cephalosporins			Carbapenems			
		**AMC**	**CTX**	**CAZ**	**FEP**	**IPM**	**AK**	**CN**	**CIP**
**P1**	Urine	R	R	R	R	R	R	R	R
**P2**	Wound	R	R	R	R	R	R	R	R
**P3**	BAL	R	R	R	R	R	S	S	R
**P4**	Burn	R	R	R	R	R	S	R	R
**P5**	Urine	R	R	R	R	R	R	R	R
**P6**	Burn	R	R	R	R	R	S	R	R
**P7**	Urine	R	R	R	S	S	S	R	R
**P8**	Urine	R	R	R	R	R	R	R	R
**P9**	Wound	R	R	R	R	R	I	R	R
**P10**	Wound	R	R	R	R	R	R	R	R
**P11**	Wound	R	R	R	R	R	R	R	R
**P12**	Wound	R	R	R	R	R	R	R	R
**P13**	Wound	R	R	R	R	R	R	R	R
**P14**	Blood	R	R	R	R	R	R	R	R
**P15**	Ear swab	R	R	R	R	I	I	R	S
**P16**	Blood	R	R	R	R	R	R	R	R
**P17**	Wound	R	R	R	R	R	R	R	R
**P18**	Blood	R	R	R	R	R	R	R	R
**P19**	Wound	R	R	R	R	S	S	S	S
**P20**	Wound	R	R	R	R	R	R	R	R
**P21**	Wound	R	R	R	R	R	S	S	S
**P22**	Urine	R	R	R	R	R	R	R	R
**P23**	Wound	R	R	R	R	R	R	R	S
**P24**	Ear swab	R	R	R	R	S	S	S	S
**P25**	Urine	R	R	S	S	I	R	I	R
**P26**	Urine	R	R	S	S	S	S	S	S
**P27**	Urine	R	R	S	S	S	S	R	R
**P28**	Urine	R	I	S	S	S	S	S	S
**P29**	Urine	R	R	S	S	S	S	I	I
**P30**	Urine	R	R	I	S	S	R	S	S

P: *Pseudomonas aeruginosa*, **BAL**: Broncho-alveolar lavage, **AMC:** Amoxicillin/clavulanic acid, **CTX:** Cefotaxime, **CAZ:** Ceftazidime, **FEP:** Cefepime, **IPM:** Imipenem, **AK:** Amikacin, **CN:** Gentamicin, **CIP:** Ciprofloxacin, **R:** Resistant, **I:** Intermediate, **S:** Sensitive

Isolate code	Clinical source	β-Lactams					Aminoglycosides		Quinolones
		Penicillin	Cephalosporins			Carbapenems			
		AMC	CTX	CAZ	FEP	IPM	AK	CN	CIP
**P31**	Urine	R	R	S	S	S	S	S	S
**P32**	Urine	R	R	S	S	S	S	S	S
**P33**	Urine	R	R	I	I	R	I	S	S
**P34**	Wound	R	R	S	S	S	S	S	S
**P35**	Wound	R	R	R	R	R	R	R	R
**P36**	BAL	R	R	R	R	R	R	R	R
**P37**	Blood	R	R	R	R	R	R	R	R
**P38**	Blood	R	R	R	R	S	S	S	I
**P39**	Blood	R	R	R	R	R	R	R	R
**P40**	Blood	R	R	R	R	R	R	R	R
**P41**	Blood	R	R	R	R	S	S	R	I
**P42**	Blood	R	R	R	R	R	R	R	R
**P43**	Blood	R	R	R	R	S	S	R	I
**P44**	Wound	R	S	R	S	S	I	R	R
**P45**	Wound	R	R	R	R	I	R	R	I
**P46**	Wound	R	R	R	R	R	R	R	R
**P47**	Wound	R	R	R	R	R	R	R	R
**P48**	Wound	R	R	R	R	R	R	R	R
**P49**	Wound	R	S	S	S	S	S	S	S
**P50**	Wound	R	R	R	R	R	R	R	R
**P51**	Wound	R	R	R	S	S	S	S	S
**P52**	Wound	R	R	R	R	R	S	R	R
**P53**	Wound	R	R	R	R	R	R	R	R
**P54**	Wound	R	R	R	R	I	S	R	R
**P55**	Wound	S	S	S	S	S	S	S	S
**P56**	Wound	R	R	R	R	R	R	R	R

P: *Pseudomonas aeruginosa*, **BAL**: Broncho-alveolar lavage, **AMC:** Amoxicillin/clavulanic acid, **CTX:** Cefotaxime, **CAZ:** Ceftazidime, **FEP:** Cefepime, **IPM:** Imipenem, **AK:** Amikacin, **CN:** Gentamicin, **CIP:** Ciprofloxacin, **R:** Resistant, **I:** Intermediate, **S:** Sensitive

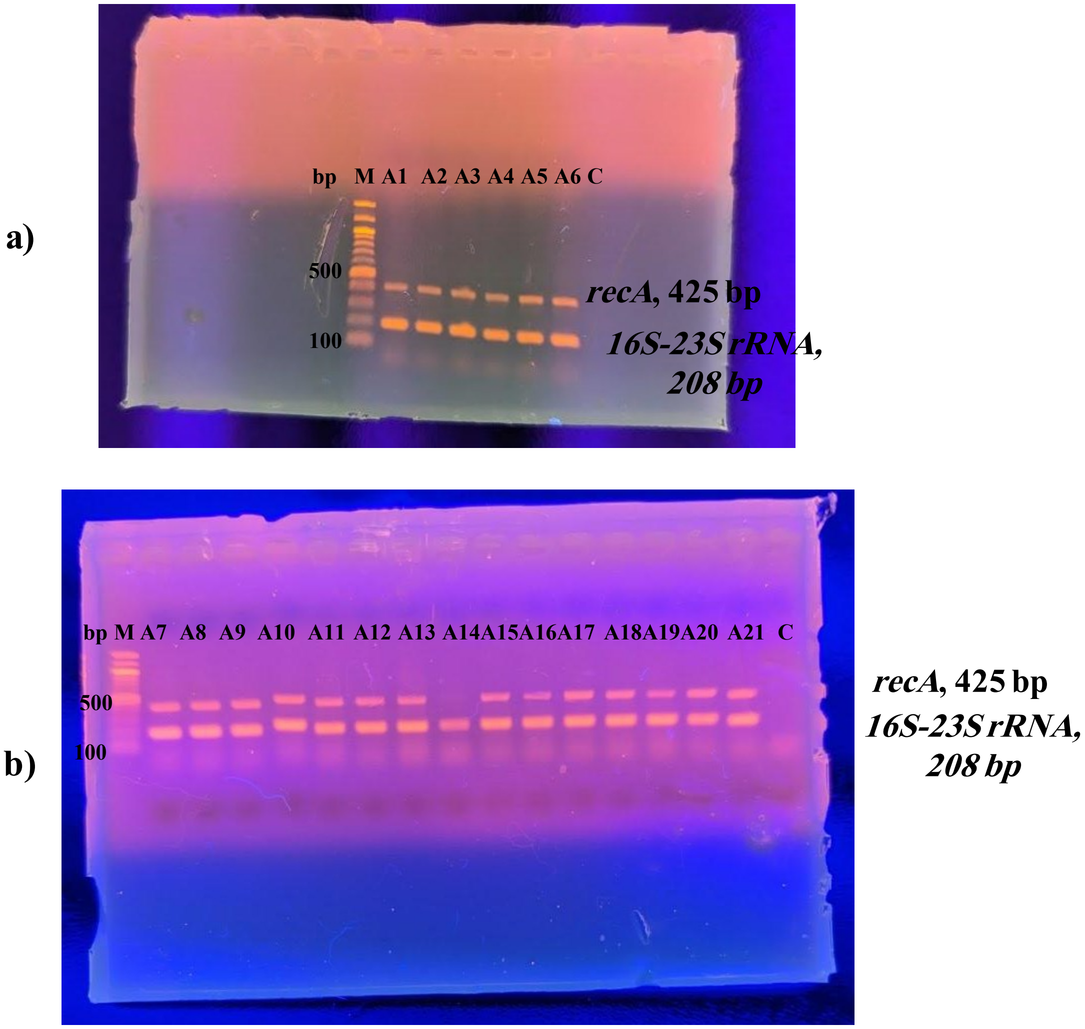

Figure S1. Agarose gel electrophoresis image of PCR amplicon of *recA* at 425 bp and *16S-23S rRNA* at 208 bp for genotypic identification of clinical *A. baumannii* isolates. Lane M: 100 bp DNA marker, C: negative control and Lanes (A1-A21): *A. baumannii* clinical isolates from A1 to A21, respectively.

a)

b)

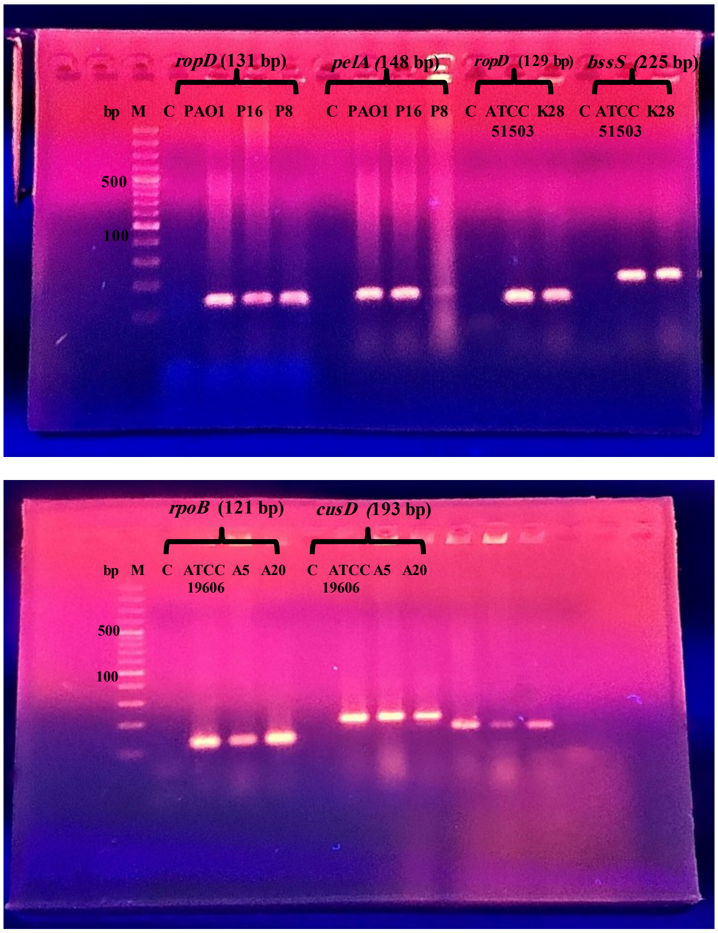

Figure S2. Agarose gel electrophoresis of (a): *P. aeruginosa* (*PAO1,* P16 and P8) (*ropD* at 131 and *pelA* at 148 bp) and *K. pneumoniae* (ATCC 51503 and K28) genes; *ropD* at 129 bp and *bssS* at 225 bp, and (b) *A. baumannii* (ATCC 19606, A5 and A20) (*rpoB* at 121 bp and *cusD* at 193 bp). Lane M: 100 bp DNA marker and C: negative control.

.
