# Supplementary figures and images for: Vitamin D and vitamin K1 as novel inhibitors of biofilm in Gram-negative bacteria
Source: BMC Microbiol. 2024 May 18;24:173. doi: 10.1186/s12866-024-03293-6 (PMC11102130; doi:10.1186/s12866-024-03293-6)

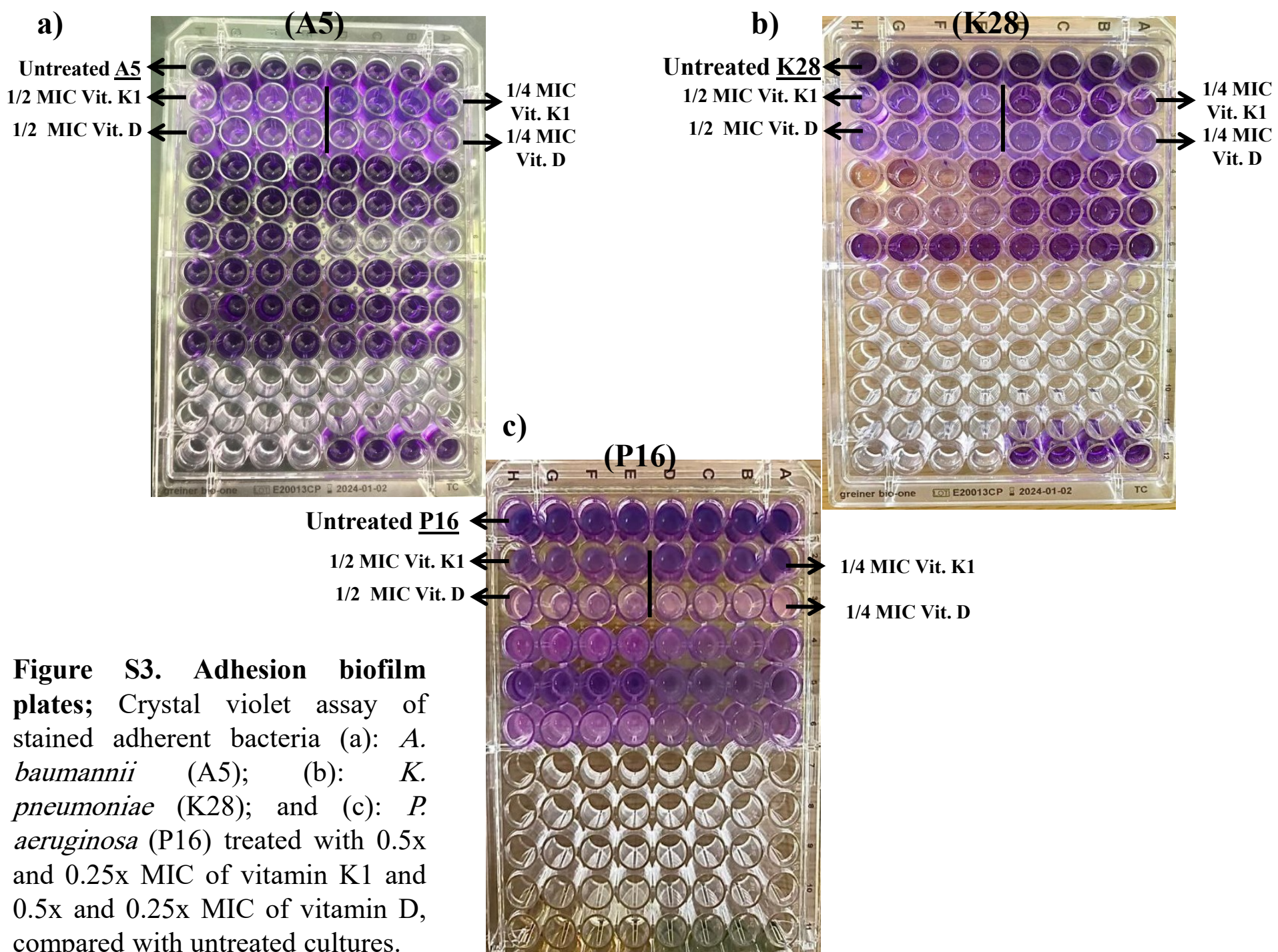

Supplement: Supplementary file 2 — Supplementary Material 2 [file 12866_2024_3293_MOESM2_ESM.pdf]
