# Supplementary material for: Vitamin D and vitamin K1 as novel inhibitors of biofilm in Gram-negative bacteria
Source: BMC Microbiol. 2024 May 18;24:173. doi: 10.1186/s12866-024-03293-6 (PMC11102130; doi:10.1186/s12866-024-03293-6)
Supplement: Supplementary file 3 — Supplementary Material 3 [file 12866_2024_3293_MOESM3_ESM.pdf]

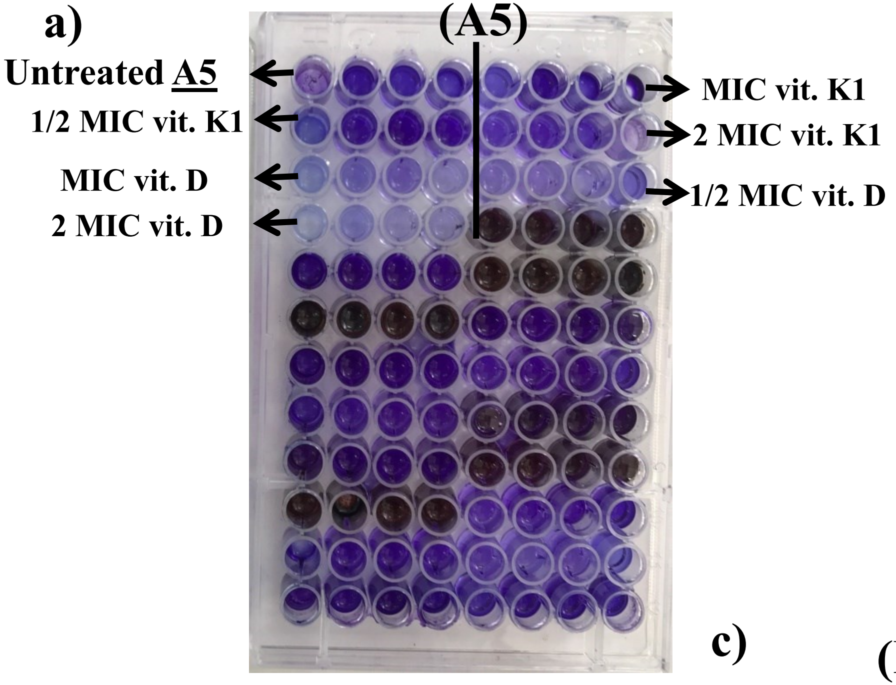

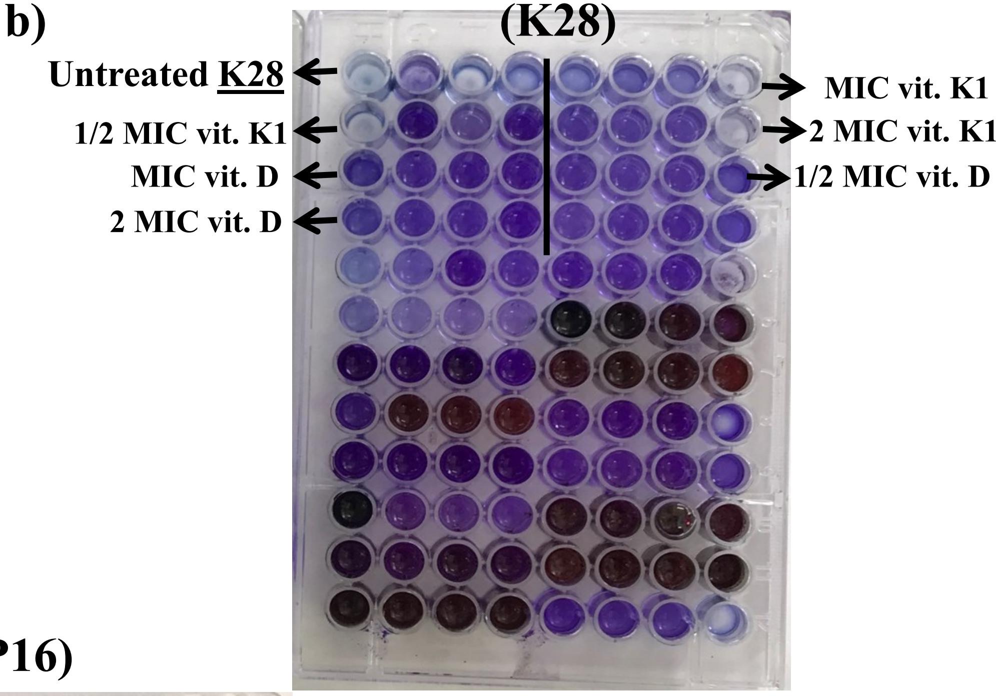

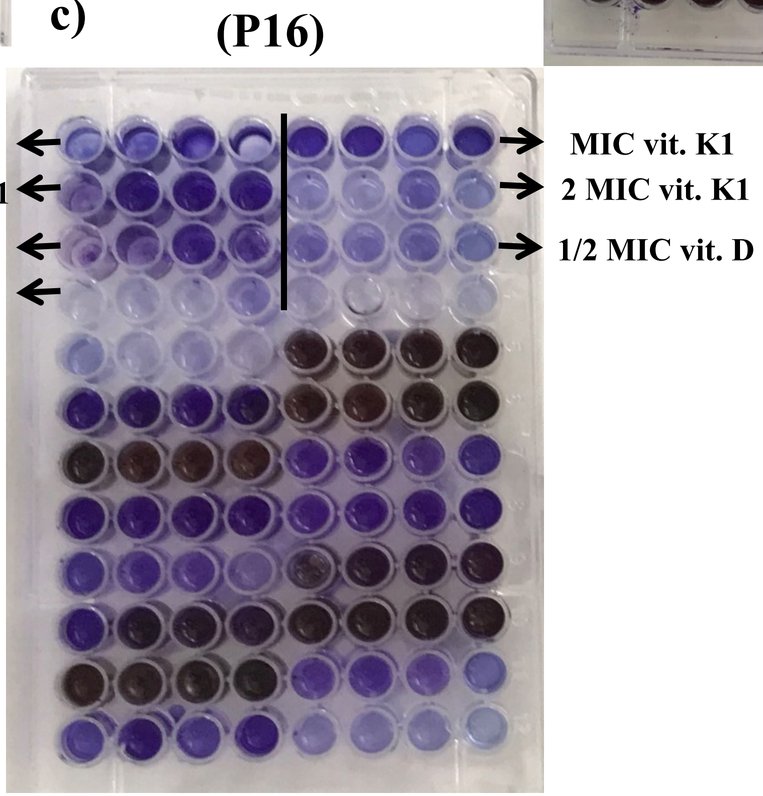

Figure S4. Mature biofilm; Crystal violet assay of stained mature biofilm formed by (a): *A. baumannii* (A5); (b): *K. pneumoniae* (K28); and (c): *P. aeruginosa* (P16) treated with 1x, 2x, 0.5x MIC of vitamin K1 and 1x, 2x, 0.5x MIC of vitamin D, compared with untreated cultures.
